# Single‐Cell Transcriptomic Profiling of Brain Cells in Newborn Rats Following Hypoxic Ischemic Encephalopathy

**DOI:** 10.1096/fj.202402891RR

**Published:** 2025-08-13

**Authors:** Xianbo Chen, Xiaohong Tao, Jingyu Wang

**Affiliations:** ^1^ Department of Pediatrics Wenling Maternal and Child Health Care Hospital Wenling Zhejiang China; ^2^ Department of Neurosurgery, the Second Affiliated Hospital Zhejiang University School of Medicine Hangzhou Zhejiang China

**Keywords:** HIE, hypoxic–ischemic encephalopathy, newborn rats, ScRNA‐seq, single cell RNA sequencing

## Abstract

Neonatal hypoxic–ischemic encephalopathy (HIE) is a severe neurological condition associated with high rates of mortality or long‐term disability. Despite its clinical significance, the detailed cellular mechanisms underlying HIE remain unclear. Single‐cell RNA sequencing (scRNA‐seq) has emerged as a powerful tool for investigating cellular heterogeneity across development, aging, and disease processes. However, no scRNA‐seq studies have yet addressed neonatal HIE. In this study, we employed scRNA‐seq to examine cellular heterogeneity during neonatal HIE. We analyzed a total of 87 580 high‐quality brain cells to identify transcriptional changes associated with HIE. In the hyperacute phase, we observed astrocytes in response to tumor necrosis factors, involvement of microglia in phagocytosis, *Stat3*‐mediated ischemic responses in oligodendrocyte precursor cells, and an increase in senescent lymphatic endothelial cells. In the acute phase, astrocytes were activated and involved in gliogenesis, while microglia proliferated. Neuroblasts were affected by metal ions, and oligodendrocytes decreased. In the subacute phase, astrocytes involved in inflammation and antigen presentation, while inflammatory microglia highly expressing MHC II were induced by the IL27 and type I interferon pathways and expanded. Additionally, peripheral immune cells played vital roles in HIE. Specifically, neutrophils infiltrated and expanded throughout all phases post‐HIE. *Spp1* ^high^ macrophages, T cells, and plasmacytoid dendritic cells increased during the acute and subacute phases, and B cells expanded during the subacute phase. This study offers deep insights into the molecular alterations of key cell types following HIE, elucidating the pathological processes involved. These findings have significant implications for developing effective clinical strategies for managing HIE.

## Graphical abstract

This study represents the first scRNA‐seq investigation revealing cellular heterogeneity during the hyperacute (3 h), acute (2 days), and subacute (7 days) phases of neonatal hypoxic–ischemic encephalopathy (HIE).

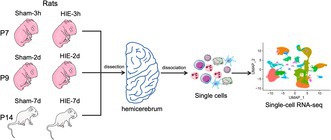

## 1 Introduction

Neonatal hypoxic–ischemic encephalopathy (HIE) is a severe neurological injury occurring after birth, leading to high rates of mortality and long‐term neurological disability [1]. It affects approximately 2–6 per 1000 newborns in developed countries and 26 per 1000 in developing countries, with significant mortality and disability rates [2, 3, 4]. Since the occurrence and development of HIE is a continuous process, the pathology of HIE progresses through three main phases [2]: i) The primary injury (0–6 h), characterized by rapid ATP depletion due to ischemia and hypoxia, leading to decreased activity of ATP‐dependent ion pumps, Ca^2+^ accumulation in neurons, and neuronal edema [4]. Excitotoxicity and lactic acid accumulation further contribute to neurotoxicity [5, 6]; ii) The secondary injury (6–72 h), marked by neuronal death shortly after reperfusion. Initial excitotoxicity exacerbates Ca^2+^ influx, triggering a cascade of events including lipase activation, mitochondrial dysfunction, nNOS activation, fatty acid release, and free radical production [2]. Mitochondrial dysfunction is central, leading to ROS accumulation, which activates microglia and astrocytes and promotes inflammation [7]. In addition, excessive ROS induces mitochondrial permeability transition pores (mPTP), facilitating the release of pro‐apoptotic factors and cytochrome c, ultimately driving apoptosis [8]; iii) The persistent injury (72 h—several weeks to years), where ongoing inflammation is predominant. This phase involves resident cells like microglia and astrocytes, as well as infiltrating immune cells such as neutrophils, monocytes, macrophages, and lymphocytes [9, 10, 11]. Persistent inflammation leads to continued neuronal death and epigenetic changes that impair oligodendrocyte maturation, axonal growth, and neurogenesis [12].

Currently, hypothermia, which lowers body and brain temperatures to approximately 32°C–34°C, is the only standard therapy for neonatal HIE. This approach offers neuroprotection by reducing ROS, free radicals, and excitatory amino acids, inhibiting inflammation, and preventing neuronal apoptosis [13, 14, 15]. Hypothermia at 33.5°C for 72 h is optimal for neonates with moderate to severe HIE; though its neuroprotective effects are limited [16, 17, 18]. Therefore, exploring additional therapies, such as drugs, cell therapy, and gene therapy, is urgent [19]. However, comprehensive understanding of the cellular mechanisms underlying HIE remains elusive, hindering the development of effective clinical targets.

Single‐cell RNA sequencing (scRNA‐seq) is emerging as a powerful technology for exploring cellular heterogeneity across development, aging, and disease processes, facilitating insights into cellular dynamics and identifying novel cell subpopulations [20, 21, 22]. ScRNA‐seq has been applied to various central nervous system injuries and diseases, including spinal cord injury, brain injury, stroke, Alzheimer's disease, and multiple sclerosis, uncovering novel injury/disease‐associated cell types [20, 23, 24, 25, 26]. However, no scRNA‐seq studies have yet addressed neonatal HIE. Given that neonatal brain cells are more naïve compared to those in adult ischemic stroke, exploring cellular heterogeneity in neonatal HIE through scRNA‐seq is warranted.

In this study, we performed scRNA‐seq to characterize cell type composition and transcriptional dynamics during the hyperacute (3 h, primary injury), acute (2 days, secondary injury), and subacute (7 days, persistent injury) phases of neonatal HIE. Our findings identified novel cell subpopulations and advanced the understanding of cellular mechanisms involved in HIE.

## 2 Methods

### 2.1 Animals

All animal experiments were performed in accordance with the China National guidelines for the care and use of laboratory animals and were approved by the Ethics Committee of Zhejiang University (No. ZJU20230229). Sprague–Dawley (SD) rats were housed at the Laboratory Animal Center of Zhejiang University with ad libitum access to sterile water and food.

### 2.2 Models of HIE

Models of HIE were established in postnatal day 7 (P7) SD rat pups, with no gender preference, following a previously described protocol with minor modifications [27]. Briefly, rat pups were randomly assigned to six groups (three males and three females per group): Sham‐3 h, HIE‐3 h, Sham‐2d, HIE‐2d, Sham‐7d, and HIE‐7d. The pups were deeply anesthetized using isoflurane inhalation. The right common carotid artery was surgically exposed and doubly ligated in the HIE groups. Sham‐operated rats underwent identical exposure of the right common carotid artery without ligation. The incision was sutured, and the pups were returned to their dams. Subsequently, the HIE rat pups were placed in a chamber with 8% oxygen for 1.5 h at 37°C.

### 2.3 Single Cell Dissociation

After 3 h, 2d, and 7d, Sham and HIE rat pups were anesthetized as described above and perfused with ice‐cold saline transcardially. Ischemic and hypoxic brains were quickly dissected and maintained in artificial cerebrospinal fluid (aCSF). Brains were cut into around 1 × 1 mm small pieces and digested in aCSF containing 1 mg/mL papain (LS003120, Washington, USA), 1 mg/mL collagen II (LS004176, Washington, USA), and 50 IU/mL DNase (EN0251, ThermoFisher Scientific, USA) for 30 min at 37°C. After digestion, a single cell suspension was generated by gentle pipette blowing and filtered through a 70 μm cell strainer. After centrifugation, cells were resuspended in 33% Percoll (17 089 109, Cytiva, Sweden) and centrifuged to remove myelin debris.

### 2.4 Single Cell RNA Sequencing

Isolated single cells were resuspended in PBS buffer at a concentration of 3 × 10^5^ cells/mL and next loaded onto a microfluidic chip (GEXSCOPE Single Nucleus RNA‐Seq Kit, Singleron, China). The preparation of scRNA‐seq libraries was conducted according to the manufacturer's protocol, and the library quantification was conducted using Qubit. Sequencing was performed on an Illumina NovaSeq 6000 instrument with 150 base pair paired‐end reads, and gene‐barcode count matrices were generated using the recommended pipeline of CeleScope. The matrices were further processed using Seurat v4.2 [28]. Cells with more than 5% mtDNA or fewer than 400 distinct genes were removed in our scRNA‐seq data. Then, sequencing data was scaled and normalized using Seurat's LogNormalize method. Highly variable genes in each cell were recognized using Seurat's FindVariableGenes function. Cell cycle status was identified using Seurat's CellCycleScoring method, and the influence of cell cycle on clustering was eliminated. Multiple scRNA‐seq matrices were integrated, and the batch correction was conducted using a Seurat CCA method. The data was next processed using a principal component analysis (PCA) method, and clustering was performed using a FindClusters method. Uniform Manifold Approximation and Projection (UMAP) was used to visualize the cell clustering. Expressed genes were calculated and identified using Seurat's FindAllMarkers function, and the differentially expressed genes (DEGs) were defined as genes with fold change > 1.5 and adjusted *p* value < 0.05. Pathway enrichment analysis (GO, KEGG pathway, WikiPathways, and Reactome Gene Sets) of DEGs was conducted using Metascape (http://metascape.org) [29]. Pseudotime analysis was performed using Monocle v3.

### 2.5 Cell Culture

The BV2 cell line (murine microglia) was obtained from the Chinese Academy of Sciences and grown in DMEM high glucose medium containing 10% FBS (Gibco, Thermo Fisher) and 1% streptomycin and penicillin. To test the phagocytosis of microglia, cells were incubated with myelin debris (10 mg/mL) originating from murine brain as previously described for 24 h [30]. Cells were collected for flow cytometry, and cell supernatant was tested for enzyme‐linked immunosorbent assays (ELISA). ELISA kits for IL‐1β (MLB00C‐1, R&D biotech, USA) and IL‐6 (M6000B‐1, R&D biotech, USA) were used to measure the concentrations of IL‐1β and IL‐6 in cell supernatant after myelin debris treatment in accordance with the manufacturer's protocol.

### 2.6 Flow Cytometry

Flow cytometric staining was conducted as previously described [31]. The cells were collected and washed twice, followed by being incubated with anti‐mouse CD16/32 antibody (Fc blocker, 156 604, BioLegend, USA) for 5 min at 4°C. The cells were then stained with APC anti‐mouse I‐A/I‐E antibody (107 613, BioLegend, USA) for 30 min at 4°C. Cells were analyzed by using a DxFLEX Flow Cytometer (Beckman, China).

### 2.7 Statistical Analysis

The data were analyzed using GraphPad Prism (10.0). Two groups of data were analyzed by Student's t tests. The data are presented as the mean ± standard error of the mean (SEM). Statistical significance was set at *p* < 0.05.

## 3 Results

### 3.1 Single‐Cell Profiling of the Brain Cells after HIE

**FIGURE 1:**
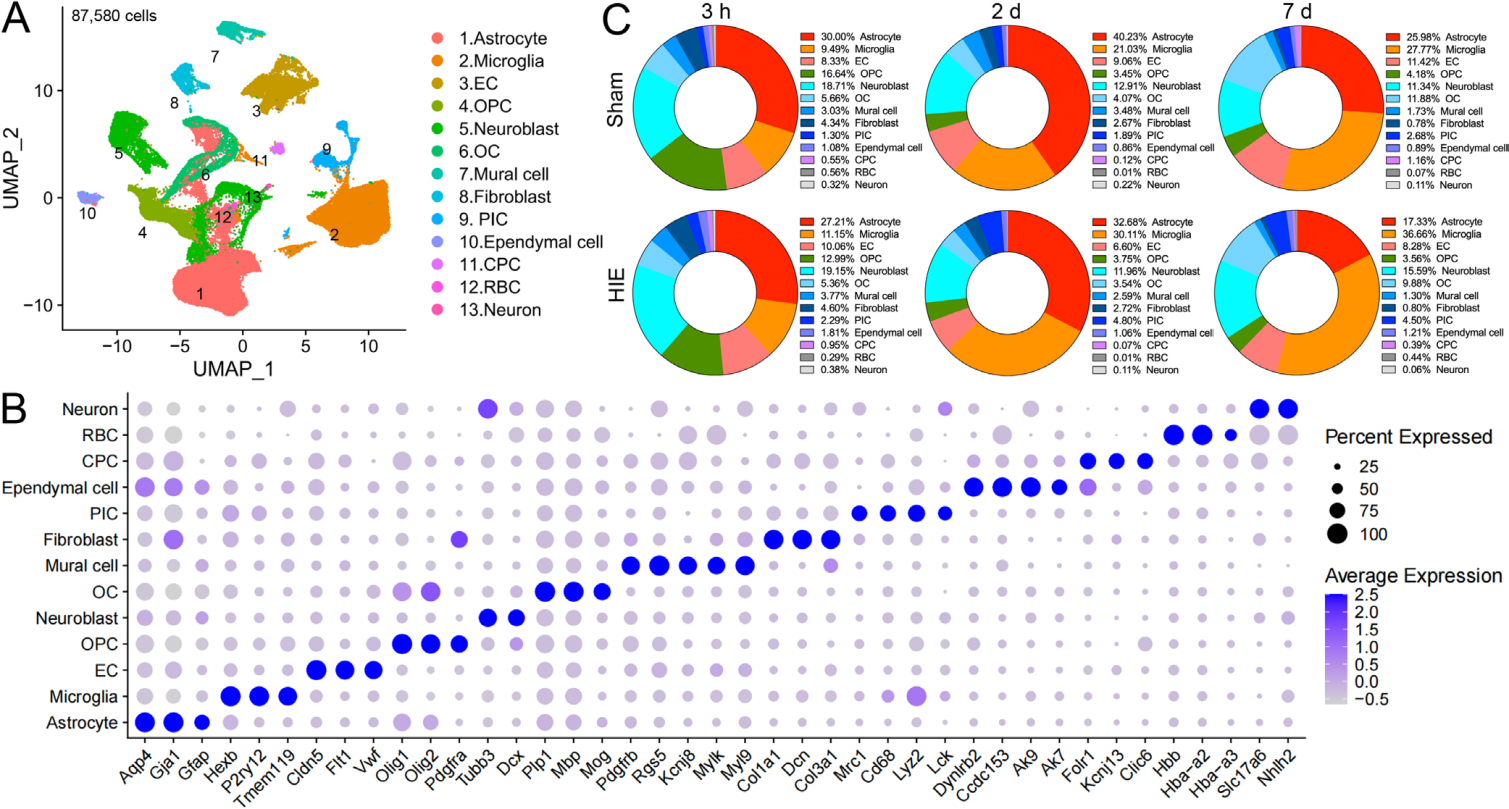
Single‐cell profiling of the brain cells after HIE. (A) A UMAP plot identifying major cell types from brains isolated from Sham and HIE rat pups. (B) A dot plot of specific cell marker genes expressed in each cell type. (C) Proportion of each brain cell type among all cells in the Sham and HIE groups. OC, oligodendrocyte; OPC, oligodendrocyte precursor cell; MP, mononuclear phagocytes; CPC, choroid plexus cell; RBC, red blood cell; EC, endothelial cells.

To investigate the cellular heterogeneity associated with HIE, scRNA‐seq was performed on brain samples isolated from HIE rat pups at three time points (3 h, 2 days, and 7 days) and their corresponding sham controls using the Singleron platform. After rigorous quality filtering, a total of 87 580 cells were analyzed, including 12 995 cells from the sham‐3 h group, 16 650 from the HIE‐3 h group, 16 364 cells from the sham‐2d group, 13 722 cells from the HIE‐2d group, 14 801 cells from the sham‐7d group, and 13 048 cells from the HIE‐7d group (Figure 1A). 13 distinct clusters were identified and visualized using a Uniform Manifold Approximation and Projection (UMAP) graph, encompassing astrocytes (25 604 cells), microglia (19 555 cells), neuroblasts (13 085 cells), endothelial cells (EC, 7917 cells), oligodendrocytes (OCs, 5828 cells), oligodendrocyte precursor cells (OPCs, 6487 cells), peripheral immune cells (PICs, 2502 cells), mural cells (2372 cells), fibroblasts (2359 cells), ependymal cells (1017 cells), choroid plexus cells (CPCs, 482 cells), red blood cells (RBCs, 193 cells), and neurons (179 cells) (Figure 1A and Table S2).

Cell type identification was dependent on established signature markers as previously described [20, 32]. Specifically, astrocytes were characterized by high expression of *Aqp4*, *Gja1*, and *Gfap*. Microglia expressed *Hexb*, *P2ry12*, and *Tmem119*. Neuroblasts were marked by *Tubb3* and *Dcx*. ECs exhibited *Cldn5*, *Flt1*, and *Vwf* expression. OCs were identified by *Plp1*, *Mbp*, and *Mog*. OPCs expressed *Pdgfra*, *Olig1*, and *Olig2*. PICs were distinguished by macrophage‐specific marker genes (*Pf4*, *Mrc1*, and *Cd68*), a monocyte‐specific marker gene (*Lyz2*), and lymphocyte‐specific marker genes (*Lck*). Mural cells expressed pericyte‐specific marker genes (*Pdgfrb*, *Rgs5*, and *Kcnj8*) and vascular smooth muscle cell‐specific marker genes (*Mylk* and *Myl9*). Fibroblasts were marked by *Dcn*, *Col3a1*, and *Col1a1*. Ependymal cells expressed *Dynlrb2*, *Ccdc153*, *Ak9*, and *Ak7*. CPCs were identified by *Folr1*, *Kcnj13*, and *Clic6*. RBCs exhibited *Hbb*, *Hbb‐a2*, and *Hbb‐a3* expression. Neurons were characterized by *Slc17a6* and *Nhlh2*. (Figure 1B and Table S1).

Doughnut diagrams illustrated the proportions of these clusters in each group (Figures 1C and S1). At the P7 stage, the Sham‐3 h group exhibited a higher proportion of naive cells, such as OPCs and neuroblasts. By P9 (Sham‐2d), the proportion of astrocytes increased, while OCs increased by P14 (Sham‐7d). During the hyperacute phase of HIE (3 h), proportions of astrocytes and OPCs decreased, whereas PICs increased. During the acute phase (2 d), the proportions of astrocytes, neuroblasts, and OCs decreased, while microglia and PICs increased. During the subacute phase (7 d), the proportions of astrocytes, ECs, and OCs decreased, while microglia, neuroblast, and PICs increased.

### 3.2 Dynamic Changes of Astrocytes Following HIE

To understand the dynamic changes in astrocytes during HIE, we initially analyzed the DEGs of astrocytes following HIE. There are 9 genes (*Ttr*, *Pf4*, *Mt1m*, *Tuba1a*, *Gfap*, et al.) upregulated in astrocytes in HIE‐3 h rats compared to Sham‐3 h rats, 29 genes (*Lgals3*, *Gfap*, *Tagln*, *Hspb1*, *C1qa*, et al.) upregulated in astrocytes in HIE‐2 d rats compared to Sham‐2d rats, and 124 genes (*Hbb*, *Mobp*, *Cd74*, *Plp1*, *Cldn11*, et al.) upregulated in astrocytes in HIE‐7 d rats compared to Sham‐7 d rats (Table S3). Enrichment pathway analysis revealed that astrocytes were related to differentiation and response to lipopolysaccharide during the hyperacute phase of HIE (Figure S2A). During the acute phase, astrocytes were associated with locomotion, immune system process, and response to stimulus (Figure S2B). During the subacute phase, astrocytes were associated with inflammatory response, regulation of gliogenesis, and antigen processing and presentation (Figure S2C).

**FIGURE 2:**
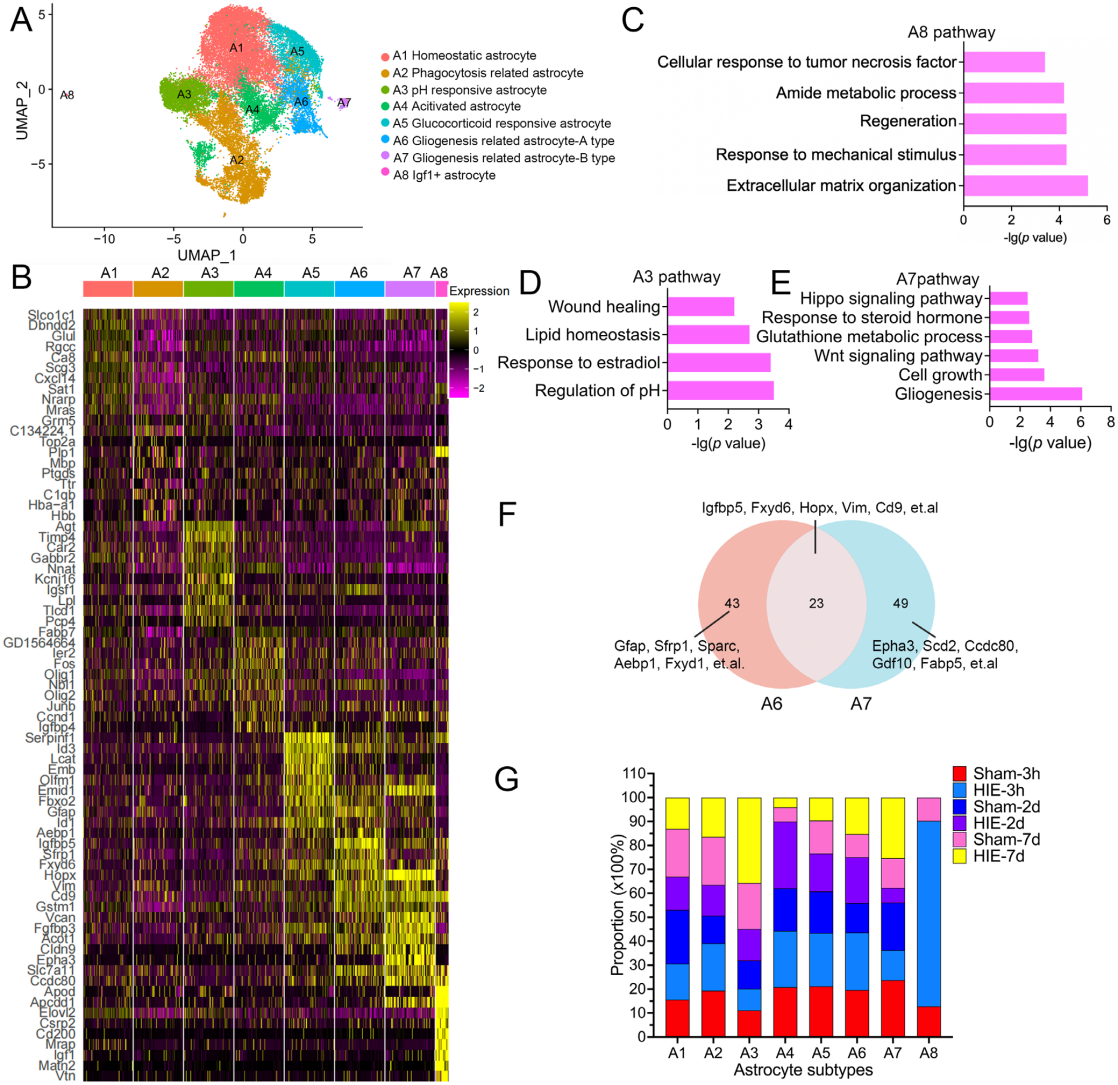
Identification of astrocyte subpopulations after HIE. (A) Re‐clustering of astrocytes visualized in a UMAP plot. (B) A heatmap plot showing the top 10 DEGs expressed in each astrocyte subpopulation. (C‐E) Pathway enrichment analysis of DEGs of A8 (C), A3 (D), and A7 (E). (F) Venn diagrams showing overlaps of upregulated DEGs of A6 and A7. (G) Proportion of each group among all astrocyte subtypes.

However, crude DEG analysis may mask the heterogeneity of astrocytes following HIE; we further performed re‐clustering of astrocytes, identifying 8 distinct subtypes (A1‐A8) (Figure 2A). DEGs analysis showed that A1 upregulated *Slco1c1*, *Dbndd2*, *Glul*, *Rgcc*, *Ca8*, *Scg3*, *Cxcl14*, et al.; A2 upregulated *Top2a*, *Plp1*, *Ptgds*, *Ttr*, *C1qb*, et al.; A3 upregulated *Agt*, *Timp4*, *Car2*, *Gabbr2*, *Nnat*, et al.; A4 upregulated *Fabp7*, *Igfbp2*, *Ier2*, *Fos*, *Nbl1*, et al.; A5 upregulated *Serpinf1*, *Id3*, *Lcat*, *Emb*, *Olfm1*, et al.; A6 upregulated *Igfbp5*, *Gfap*, *Sfrp1*, *Fxyd6*, *Hopx*, et al.; A7 upregulated *Hopx*, *Vcan*, *Cd9*, *Fgfbp3*, *Emid1*, et al.; A8 upregulated *Apod*, *Apcdd1*, *Elovl2*, *Mia*, *Plp1*, et al. (Figure 2B and Table S4). According to a previous study [33], gene expression and pathway analysis (Figures 2C–E and S3), A1 was defined as homeostatic astrocyte. A2 upregulated other cell type markers such as *Plp1*, *Mbp*, *C1qb*, *Hbb*, et al. which were defined as phagocytosis‐related astrocytes (Figure 2B). A3 was associated with regulation of pH, which was recognized as pH‐responsive astrocyte (Figure 2D). A4 upregulated *Igfbp4* and immediate early genes (IEGs) including *Egr1*, *Fos*, and *Junb*, which were defined as activated astrocytes (Figure 2B). A5 was associated with regulation of growth and response to glucocorticoids, which were defined as glucocorticoid‐responsive astrocytes (Figure S3A). A6 and A7 were related to gliogenesis, which were defined as gliogenesis‐related astrocytes (Figures 2E and S3B). A8 was recognized as *Igf1* ^+^ astrocyte (Figure 2B). A Venn plot showed 23 core genes (*Igfbp5*, *Fxyd6*, *Hopx*, *Vim*, *Cd9*, et al.) for gliogenesis‐related astrocytes, 43 genes (*Gfap*, *Sfrp1*, *Sparc*, *Aebp1*, *Fxyd1*, et al.) specific for gliogenesis‐related astrocyte‐A type, and 49 genes (*Epha3*, *Scd2*, *Ccdc80*).

*Gdf10*, *Fabp5*, et al.) specific for gliogenesis‐related astrocyte‐B type (Figure 2F). Enrichment pathway analysis showed that gliogenesis‐related astrocyte‐A type was related to the MAPK signaling pathway, while gliogenesis‐related astrocyte‐B type was related to the Wnt signaling pathway (Figure S4A,B).

Figure 2G illustrates the proportion of each group among all astrocyte subtypes (Figure S4C and Table S2). Notably, A8 was elevated in rats at 3 h post‐HIE compared to Sham P7 rats (77.49% vs 12.81%). GO pathway enrichment analysis showed that A8 was associated with extracellular matrix organization (*Pmp22*, *Nid1*, *Col11a1*, *Col12a1*, *Vtn*, *Ccdc80*, *Mia*, *Col28a1*, and *Postn*), regeneration, and amide metabolic process (*Gsta1*, *Hmgcs2*, *Dbi*, *Mgst1*, *Bace2*, *Gsta4*, *Gstt3*, *Elovl1*, and *Gal3st1*). In addition, A8 may be mediated by the TNF signaling pathway with the high expression of *Nfkbia*, *Ctsk*, *Mapk3*, *Akap12*, *Postn*, and *Tnbs1* (Figure 2C). Activated astrocytes (A4) increased at 2 days post‐HIE (27.87% vs 17.77%). A3 and A7 were more prevalent in mice at 7 days post‐HIE compared to sham controls (35.68% vs 19.21% and 25.36% vs 12.31%, respectively) (Figure 2G). Pathway enrichment analysis showed that A3 was related to regulation of *PH*, response to estradiol, lipid homeostasis, and wound healing. Furthermore, A7 exhibited increased expression of cell growth‐related genes (*Agt*, *Ddr1*, *Nfix*, *Nrp2*, and *Ndn*), Wnt signaling pathway‐associated genes (*Ccnd2*, *Lef1*, *Wnt8b*, *Frzb*, and *Apcdd1*), and Hippo signaling pathway (*Ywhaq*, *Ccnd2*, *Lef1*, and *Wnt8a*).

### 3.3 Dynamic Changes of Microglia Following HIE

**FIGURE 3:**
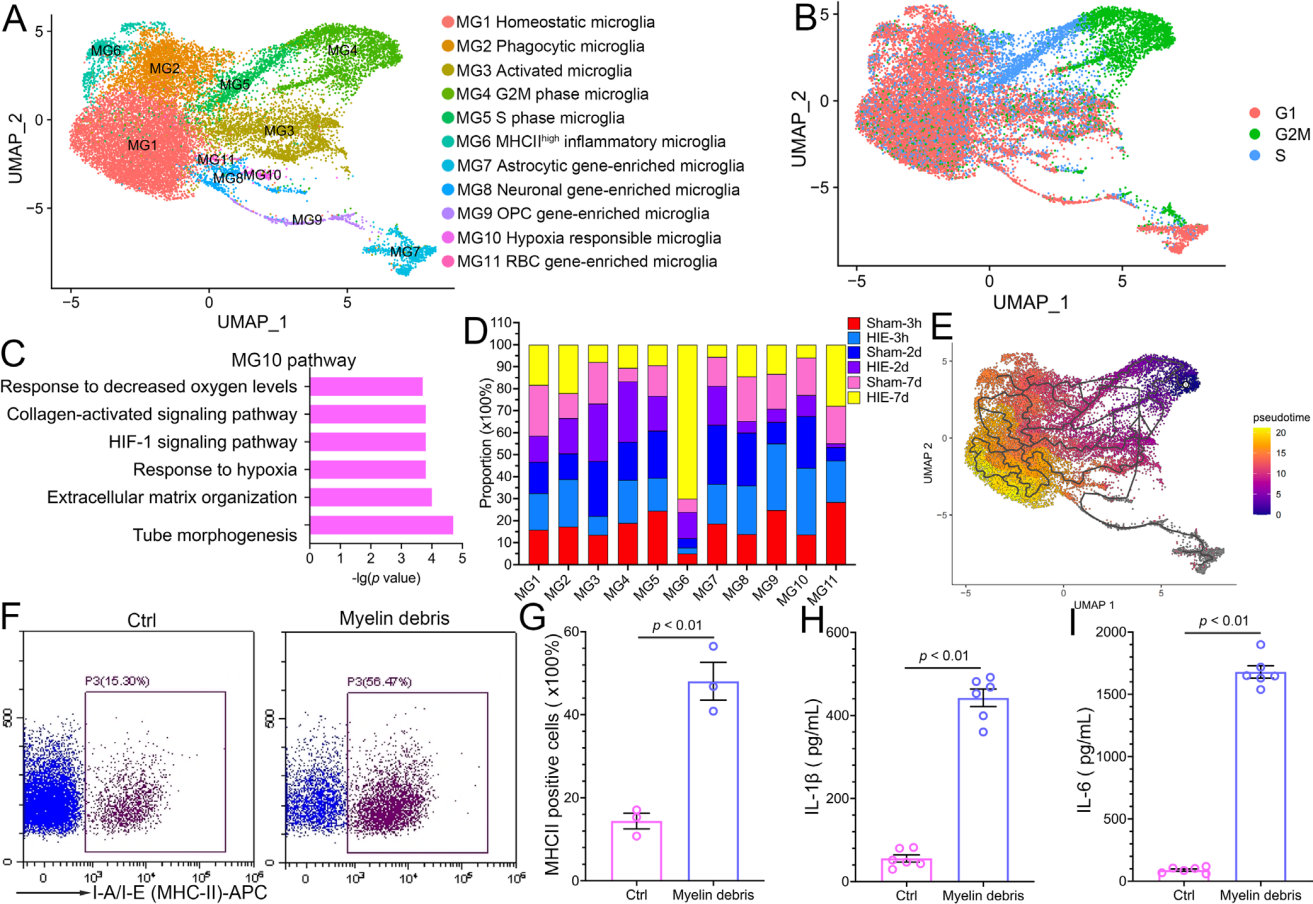
Identification of microglial subpopulations after HIE. (A) Re‐clustering of microglia visualized in a UMAP plot. (B) A UMAP plot showing cell cycle analysis of microglia. (C) Pathway enrichment analysis of DEGs of MG10. (D) Proportion of each group among all microglial subtypes. (E) A UMAP plot showing the single cell trajectory of microglia using Monocle v3. Cells are ordered in pseudo time colored in a gradient from purple to yellow. (F) Flow cytometry showing MHC II positive microglia with or without incubating with myelin debris. (G) Statistical graph of F. (H and I) ELISA assays showing the secretion of IL‐1β (H) and IL‐6 (I) in microglia with or without incubating with myelin debris.

Microglia were subsequently collected and re‐clustered into 11 subtypes (MG1‐M11) (Figure 3A). According to DEGs (Figure S5 and Table S5), MG1 expressed high levels of homeostatic microglia marker genes such as *Tmem119*, *Sparc*, *P2ry12*, *Siglec5*, *Hexb*, and *Cx3cr1*, classifying it as homeostatic microglia [20, 34]. MG2 upregulated phagocytic genes such as *Cd68* and lysosome‐related genes such as *Cd63*, *Ctsb*, *Atp6v0c*, *Ctsd*, *Dnase2*, *Ctsz*, *Npc2 Ctsa*, and *Hexa*, defining it as phagocytic microglia (Figure S5) [35]. We defined MG3 as activated microglia highly expressing IEGs such as *Fos* and *Egr1*. Cell cycle analysis identified MG4 as G2M phase microglia and MG5 as S phase microglia (Figure 3B). Notably, MG6 upregulated MHC II‐related genes (*Cd74*, *RT1‐Db1*, *RT1‐Da*, *RT1‐Ba*, *RT1‐Bb*, et. al) and inflammatory genes (*C3*, *Cxcl16*, *Ccl6*, *Cxcl2*, *Ccrl2*, *Ccl4*, et al.), which were defined as MHC II^high^ inflammatory microglia. Some microglial subtypes (MG7‐9, MG11) co‐expressed other cell type marker genes. MG7 was enriched with astrocytic genes (*Aqp4*, *Gja1*, *S100b*, *Gfap*, *Sox9* et al.), MG8 expressed neuronal genes (*Tubb3*, *Stmn2*, *Dcx*, *Map2*, *Map1b*, et al.) with oligodendroglia genes (*Mog*, *Mag*, *Mobp*, *Mbp*, *Plp1*, et al.), MG9 had OPC specific genes (*Scrg1*, *Olig1*, *Olig2*, *Sox10*, *Pdgfra*, et al.), MG11 had RBC specific genes (*Hbb*, *Hba‐a1*, *Hba‐a2*, *Hba‐a3*, et al.) In addition, MG10 upregulated hypoxia‐associated genes such as *Slc2a1*, *Kdr*, *Plat*, *Epas1*, *Flt1*, and *Tfrc*, which were defined as hypoxia‐responsible microglia (Figure 3C).

The UMAP distribution of each sample for each microglial subcluster category is shown in Figure S6A. The quantity of microglia increased during development, remaining stable during the hyperacute phase of HIE but rising during the acute and subacute phases. Figure 3D depicts the proportions of each microglial subtype over time. Homeostatic microglia (MG1) decreased during HIE (HIE‐2d: 14.3% vs 11.89%, HIE‐7d: 23.07% vs 18.37%). Phagocytic microglia (MG2) decreased with development (Sham‐3 h (P7): 17.22%, Sham‐2d (P9): 11.71%, Sham‐7d (P14): 11.26%), but increased after HIE (HIE‐3 h: 21.6%, HIE‐2d: 16.1%, HIE‐7d: 22.11%), especially during the subacute phase. Notably, hypoxia responsible microglia (MG10) increased following HIE at 3 h compared to their corresponding Sham group (30.24% vs 13.66%). The proportion of G2M phase microglia increased in the HIE‐2d group compared to their corresponding Sham group (27.39% vs 17.43%). MHC II^high^ inflammatory microglia (MG6) reached up to 70.02% at 7 days post‐HIE. Pathway analysis further revealed that MG6 was related to antigen processing and presentation via MHC class II and innate immune response (Figure S6B).

CytoTRACE analysis predicted MG4 as the starting point of the microglial single‐cell trajectory path (Figure S6C). Monocle (v3) analysis suggested that MG6 may originate from MG2 (phagocytic microglia) (Figure 3E). To confirm this origination, BV2 (a microglial cell line) was treated with myelin debris for 24 h. Flow cytometry staining and ELISA showed that BV2 significantly upregulated IA/IE (MHC II) and inflammatory cytokines (IL‐1β and IL‐6) after engulfing myelin debris (Figure 3F–I). Inflammatory microglia (MG6) were prominent in the HIE‐7d group, potentially crucial for HIE progression.

### 3.4 Dynamic Changes of Neuroblast and Oligodendrocyte Lineage Following HIE

**FIGURE 4:**
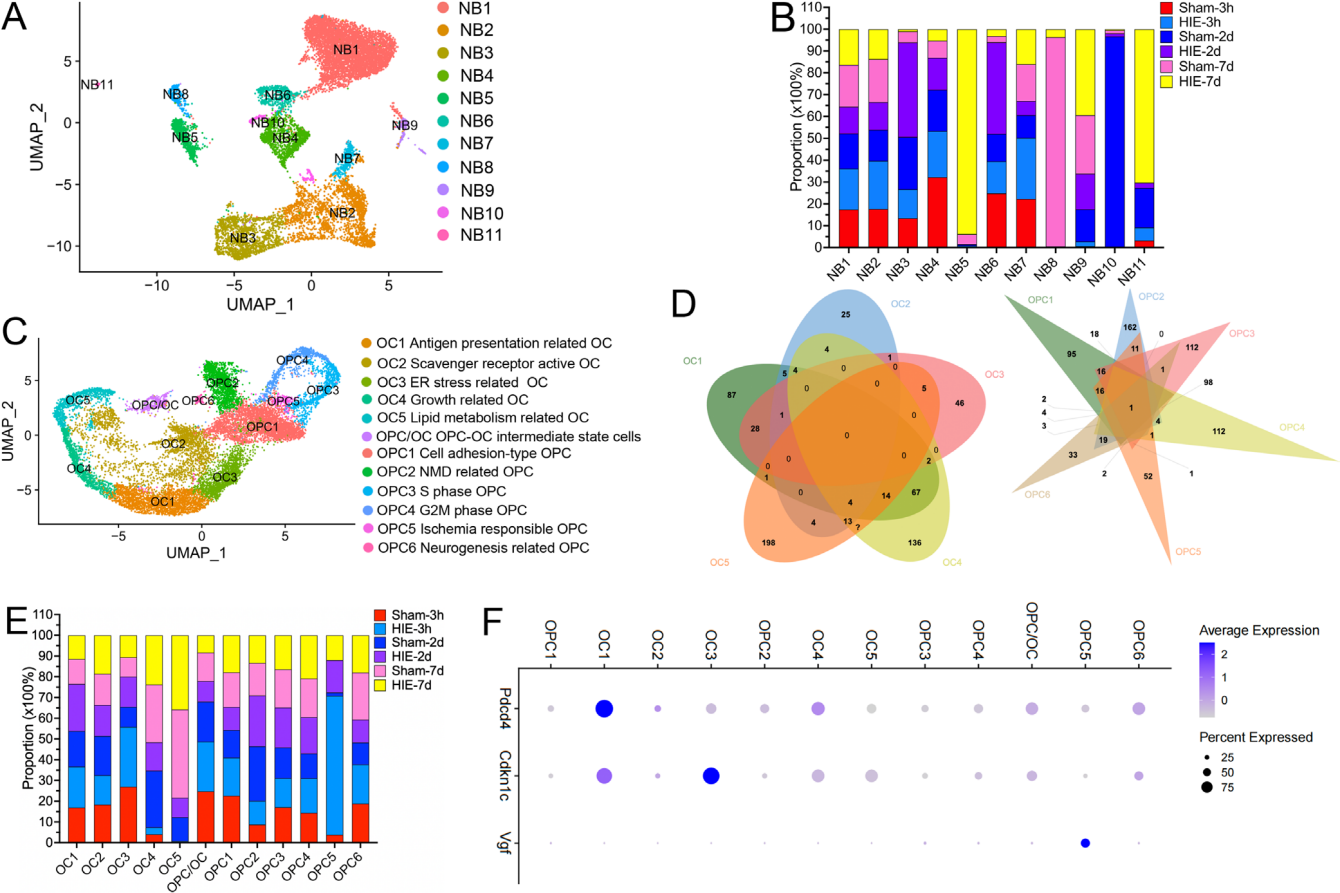
Identification of neuroblast and oligodendrocyte lineage subpopulations after HIE. (A) Re‐clustering of neuroblasts visualized in a UMAP plot. (B) Proportion of each group among all neuroblast subtypes. (C) Re‐clustering of oligodendrocyte lineage visualized in a UMAP plot. (D) Venn diagrams showing overlaps of DEGs of oligodendrocyte subtypes and OPC subtypes. (E) Proportion of each group among all oligodendrocyte lineage subtypes. (F) A dot plot showing the expression of *Pdcd4*, *Cdkn1c*, and *Vgf* in each oligodendrocyte lineage subpopulation.

Re‐clustering of neuroblasts was performed to elucidate the dynamic changes in neuroblasts following HIE. We successfully obtained a total of 11 subclusters of neuroblasts (NB1‐12) (Figure 4A). Specifically, DEGs analysis (Figure S7A and Table S6) revealed that NB1 exhibited high expression of *Dlx1*, *Tlam2*, *Stmn2*, et al. NB2 upregulated *Top2a*, *Hig5*, *Ube2c*, *Mki67*, *Cenpf*, et al. NB3 upregulated *Slc1a3*, *Igfbp2*, *Ptprz1*, *Aqp4*, *Mt3*, et al. NB4 upregulated *Sema3c*, *Nfix*, et al. NB5 upregulated *Itm2a*, *Ptgds*, et al. NB6 upregulated *Reln*, *Mef2c*, *Syt1*, et al. NB7 upregulated *Ccnd2*, *Mcm5*, *Rgs2*, *Cdca7*, et al. NB8 upregulated *Gngt1*, *Tph1*, *Camk2b*, *Gngt2*, *SLc17a7*, et al. NB9 upregulated *Aif1*, *Tmem176b*, et al. NB10 upregulated *Selenom*, *Mab21l1*, et al. NB11 upregulated *Igf1*, *Mrap*, *Frzb*, et al.

The proportion of NB3 increased during the acute phase of HIE (43.23% vs. 23.98%) (Figures 4B, S7B). Pathway analysis of DEGs revealed that NB3 was associated with cell division and response to copper ion and fatty acid (Figure S7C). NB5 and NB11 were increased during the subacute phase of HIE (Figures 4B, S7B). Pathway analysis of DEGs revealed that NB5 was related to cell activation, MAPK cascade, response to metal ion, and inflammation. In addition, NB11 was associated with lipid metabolism (Figure S7D).

To further investigate the dynamic changes in oligodendrocyte lineage cells following HIE, OPCs and OCs were re‐clustered into 12 subclusters, including five subtypes of OCs, six subtypes of OPCs, and one intermediate OC/OPC subtype (Figure 4C). Based on significant DEGs identified in each subcluster and subsequent enrichment pathway analyses (Figures 4C,D,S8A, Tables S7‐, S9), the subclusters were characterized as follows: OC1 as antigen presentation‐related OCs, OC2 as scavenger receptor‐active OCs, OC3 as ER stress‐related OCs, OC4 as growth‐related OCs, OC5 as lipid metabolism‐related OCs, OPC/OC as intermediate state OPC‐OC cells, OPC1 as cell adhesion‐type OPCs, OPC2 as NMD‐related OPCs, OPC3 as S‐phase OPCs, OPC4 as G2M‐phase OPCs, OPC5 as ischemia‐responsible OPCs, and OPC6 as neurogenesis‐related OPCs (Figure 4D). It was observed that OPCs progressively transitioned into OCs during development (Figure S8B). During the acute and subacute phases of HIE, the number of OCs decreased while the number of OPCs remained unchanged, suggesting that HIE may lead to myelin damage or impairment of myelin maturation. Notably, a significant enrichment of ischemia‐responsible OPCs was observed following HIE, particularly during the hyperacute phase (HIE3h‐67.05%; HIE2d‐15.57%; HIE7d‐11.98%) (Figures 4E, S8B). Ischemia‐responsible OPCs upregulated *Vgf* (Figure 4F). Interaction network analysis of DEGs suggested that *Stat3* might be a critical transcription factor for ischemia‐responsible OPCs (Figure S9). In addition, the proportions of *Pdcd4* ^high^ OC1 and *Cdkn1c* ^high^ OC3 slightly increased during the acute phase of HIE (22.75% vs. 17.17%, 14.55% vs. 9.67%, respectively) (Figure 4F).

### 3.5 Dynamic Changes of Endothelial Cells and Fibroblasts Following HIE

**FIGURE 5:**
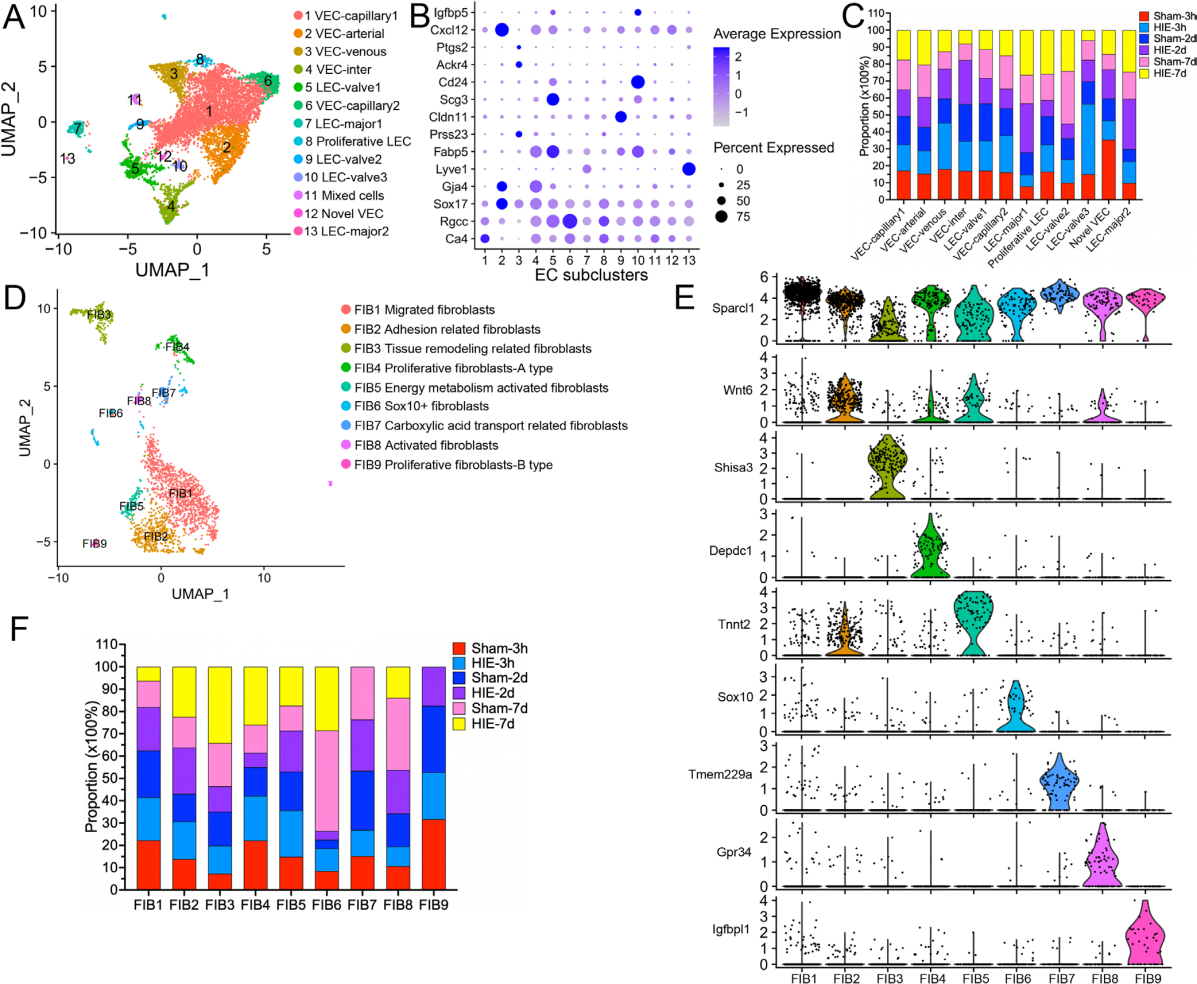
Identification of endotheliocyte and fibroblast lineage subpopulations after HIE. (A) Re‐clustering of VEC visualized in a UMAP plot. (B) A dot plot showing specific cell marker genes expressed in each endotheliocyte subtype. (C) Proportion of each group among all endotheliocyte subtypes. (D) Re‐clustering of fibroblasts visualized in a UMAP plot. (E) Violin plots showing certain genes expressed in each fibroblast subtype. (F) Proportion of each group among all fibroblast subtypes.

Re‐clustering of endothelial cells was conducted to explore dynamic changes of these cells following HIE. 13 subpopulations were identified, including six subclusters of vascular endothelial cell (VEC), five subclusters of lymphatic endothelial cell (LEC), and one subcluster of mixed cells that were identified (Figure 5A). According to a previous study and DEGs (Figures 5B and S10A, Table S10) [36], we identified *Ca4* ^high^ VEC‐capillary that also highly expresses *Nrgn*, *Tfrc*, *Slc22a8*, and *Hmcn1*, and *Rgcc* ^high^ VEC‐capillary that highly expresses *Aqp4*, *Ptprz1*, *Slc1a3*, *Mt3*, and *Fabp7*. In addition, we identified *Sox17* ^high^ *Gja4* ^high^ VEC‐arterial, *Mdcam1* ^high^ VEC‐venous, VEC‐inter that co‐expresses VEC‐arterial and VEC‐venous marker genes, and a novel VEC subpopulation that highly expresses *Dcn*, *Slc7a11*, *Igf2*, *Col1a1*, and *Ptgds*. For LEC, we identified *Lyve1* ^low^ LEC‐major, *Lyve1* ^high^ LEC‐major, *Scg3* ^high^ LEC‐valve, *Cldn11* ^high^ LEC‐valve, and *Cd24* ^high^ LEC‐valve. We noticed that the proportion of LEC‐valve3 increases during the hyperacute phase of HIE (41.49% vs. 15.01%) (Figures 5C,S10B). Enrichment pathway analysis of significant DEGs in LEC‐valve3 showed that these LECs were independently related to cellular senescence with the high expression of *Jun*, *Cdkn1b*, *Cdk4*, *Ube2s*, *Ube2c*, *Ezh2*, *Fos*, *H2ax*, and *H2az2* (Figure S10C).

Fibroblasts were also re‐clustered into nine subtypes (FIB1‐9) (Figure 5D). FIB1 was defined as migrated fibroblasts, expressing *Sparcl1*; FIB2, as adhesion‐related fibroblasts, expressing *Wnt6*; FIB3 as tissue remodeling‐related fibroblasts, expressing *Tnnt2*; FIB4 and FIB9, as proliferative fibroblasts, with FIB4 expressing *Depdc1* and FIB9 expressing *Igfbpl1*; FIB5, as energy metabolism‐activated fibroblasts, expressing *Tnnt2*; FIB6 expressing *Sox10*; FIB7, as fibroblasts related to carboxylic acid transport, expressing *Tmem229a*; and FIB8, as activated fibroblasts, expressing *Gpr34* (Figure 5E and Table S11). Changes in fibroblast proportions were observed primarily during the subacute phase of HIE, with increases in FIB2‐5 (22.47% vs. 13.79, 34.16% vs. 19.36%, 26.01% vs. 12.52%, 17.37% vs. 11.29%, respectively) (Figures 5F,S10D).

### 3.6 Dynamic Changes of Immune Cells Following HIE

**FIGURE 6:**
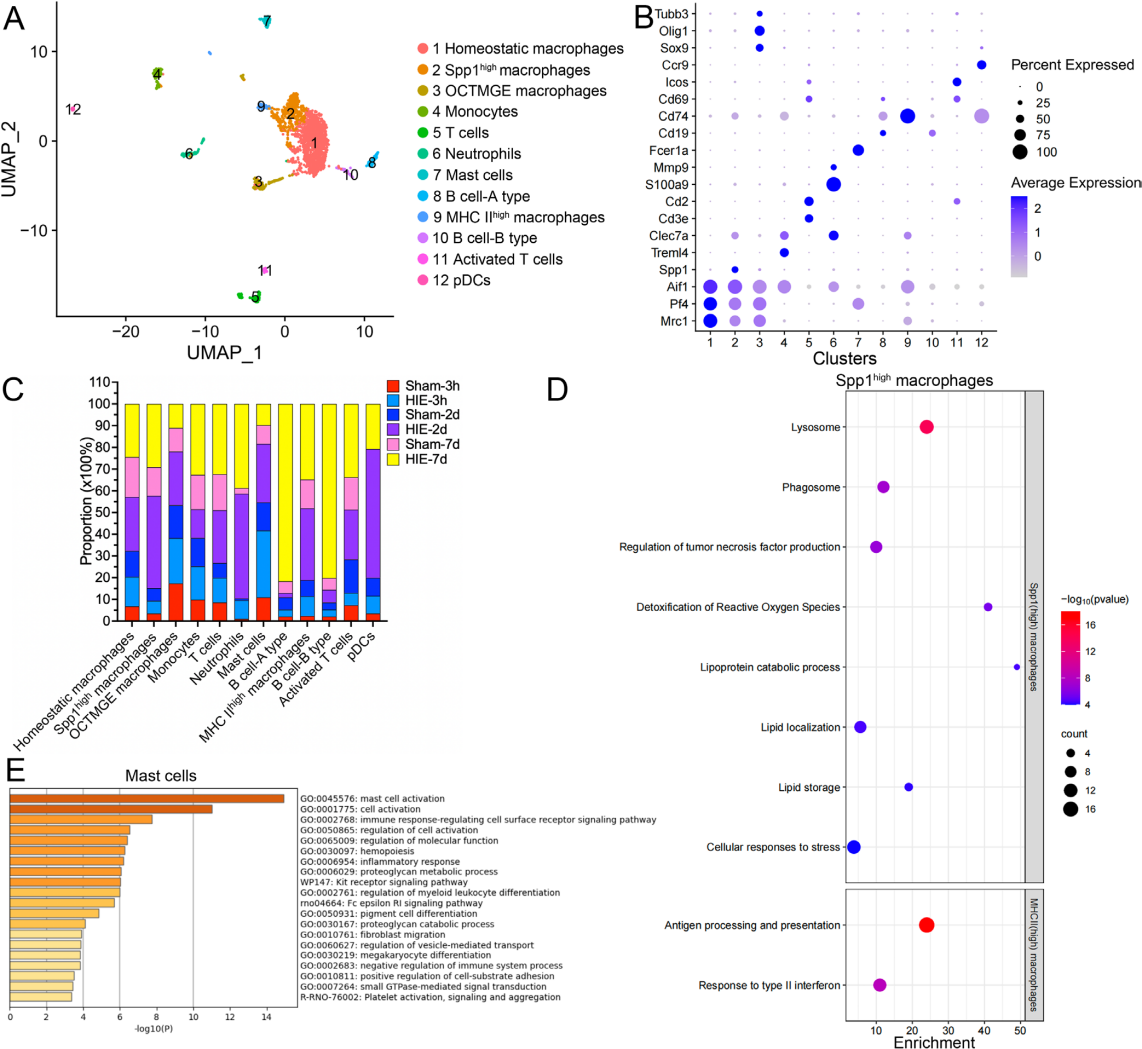
Identification of immune cell subpopulations after HIE. (A) Re‐clustering of infiltrated immune cells visualized in a UMAP plot. (B) A dot plot showing specific cell marker genes expressed in each infiltrated immune cell subtype. (C) Proportion of each group among all infiltrated immune cell subtypes. (D) A dot plot showing pathway enrichment analysis of DEGs of *Spp1* ^high^ macrophages. (E) Pathway enrichment analysis of DEGs of mast cells.

Re‐clustering of the PIC compartment revealed the presence of various immune cell types, including neutrophils, mast cells, B cells, T cells, and dendritic cells (DCs), in addition to macrophages and monocytes (Figure 6A). These cells were classified as infiltrated peripheral immune cells. Macrophages specifically expressed markers such as *Mrc1*, *Pf4*, and *Aif1* (Figure 6B). Other cell type marker genes enriched (OCTMGE) macrophages upregulated astrocyte, oligodendrocyte, and neuron marker genes such as *Sox9*, *Olig1*, and *Tubb3*. MHCII^high^ macrophages upregulated *Cd74*. Monocytes specifically expressed *Treml4*. T cells specifically expressed *Cd3e* and *Cd2*. Activated T cells upregulated activated marker genes including *Cd69* and *Icos*. Neutrophils specifically expressed *S100a9* and *Mmp9*. Mast cells specifically expressed *Fcer1a*. B cells specifically expressed *Cd19*. Plasmacytoid dendritic cells (pDCs) specifically expressed *Ccr9*.

We identified four macrophage subtypes: homeostatic macrophages highly expressed macrophage marker genes, including *Aif1*, *Pf4*, and *Mrc1*, whereas OCTMGE macrophages, *Spp1* ^high^ macrophages, and MHCII^high^ macrophages down‐regulated these marker genes. The proportion of OCTMGE macrophages increased 2 days after HIE (24.64% vs. 15.25%), which may indicate that these macrophages phagocytose other cells containing these mRNA (Figures 6C,S11A). Importantly, *Spp1* ^high^ macrophages increased during acute and subacute phases of HIE (Acute, 42.43% vs. 5.9%; Subacute, 29.13% vs. 13.25%) (Figure 6C), which was related to lysosome, phagosome, TNF production, detoxification of reactive oxygen, lipid metabolism, and response to stress (Figure 6D). MHCII^high^ macrophages increased following HIE (Hyperacute, 9.09% vs. 2.33%; Acute, 33.09% vs. 7.4%; Subacute, 34.8% vs. 13.29%) (Figure 6C), which was related to antigen processing and presentation and response to IFN‐γ (Figure 6D). Furthermore, neutrophils infiltrated and were expanded in the brain following HIE (Hyperacute, 8.57% vs. 1%; Acute, 48.22% vs. 0.79%; Subacute, 38.78% vs. 2.63%). Mast cells increased during hyperacute and acute phases of HIE (Hyperacute, 30.71% vs. 10.93%; Acute, 26.91% vs. 13.02%), which was associated with mast cell activation, inflammation, proteoglycan metabolic process, and fibroblast migration (Figure 6E). T cells and pDCs increased during acute and subacute phases of HIE. Two subtypes of B cells were discovered in our data. Significant DEGs of B cell‐A type was related to the regulation of B cell proliferation, formation of a pool of free 40S subunits, and regulation of calcium ion transmembrane transport, whereas B cell‐B type was associated with metabolism of RNA, cell cycle, and B cell activation (Figure S11B). However, these two types of B cell merely increased at the subacute phase of HIE (A‐type, 81.7% vs. 5.4%; B‐type, 80.13% vs. 5.43%).

## 4 Discussion

This study represents the first scRNA‐seq investigation revealing cellular heterogeneity in neonatal HIE. In the hyperacute phase, astrocytes are activated in response to TNF, microglia engage in phagocytosis, and there is an increase in *Stat3*‐induced ischemia‐responsible OPCs and senescent LECs. In the acute phase, microglia proliferate, neuroblasts are affected by metal ions, and oligodendrocytes decrease. During the subacute phase, astrocytes facilitate tissue repair, and inflammatory microglia expand with elevated MHC II expression. Metal ion‐responsible neuroblasts and fibroblasts increase, while OCs further decrease. In addition, peripheral immune cells are involved throughout HIE. Neutrophil infiltrate and expand in the brain across all phases; *Spp1* ^high^ macrophages, T cells, and pDCs increase during the acute and subacute phases, and B cells expand during the subacute phase.

Astrocytes play a critical role in synaptic development and function in the brain [37]. HIE induces dynamic changes in astrocytes. We noticed that A10 was predominantly discovered during the hyperacute phase (3 h). Notably, *Igf1*, encoding insulin‐like growth factor 1 (IGF‐1), was significantly upregulated in A10. In vitro experiments have demonstrated that astrocytes expressed high levels of IGF‐1 in response to TNF‐α stimulation facilitating myelination [38]. This result and our pathway analysis revealed that the inflammatory factor TNF may promote the generation of this astrocyte subtype. During the acute phase (2 d), astrocytes are activated in response to ischemia and hypoxia, exhibiting high expression of IEGs. These activated astrocytes facilitate translation processes. Notably, *Igfbp4* emerged as a specific marker for these activated astrocytes. *Igfbp4* encodes IGFBP4, which binds and inhibits IGF‐I/II, with its levels regulated by PAPP‐A dependent proteolysis [39]. The IGF pathway is essential for organismal development, and IGF1 knockout in mice leads to microcephaly [40]. HIE‐induced IGFBP4^high^ astrocytes may inhibit brain development, and targeting IGFBP4 may be a potential therapy in the early stage. During the subacute phase (7 d), astrocytes are converted to a repair phenotype, contributing to wound healing.

During central nervous system (CNS) development, substantial remodeling occurs through synaptic pruning, leading to a precise neural circuit wiring diagram [41, 42]. Microglia contribute to synaptic plasticity by phagocytosing and eliminating excess dendrites, axons, and synapses generated during development [43, 44]. Our data indicate that phagocytic microglia are particularly prevalent in the early developmental stage (P7) and their numbers increase following HIE, likely to clear injured cell debris. HIE also induces microglial proliferation, though to a lesser extent than in other CNS injuries [20, 45]. However, a lower degree of microglial proliferation was induced via HIE compared to CNS injury. We speculate about the possible reasons, including: i) microglia in the developmental stage owe proliferative characteristics, and the proliferative potential is weak, while most adult microglia are homeostatic, and the proliferative potential is stronger; ii) CNS injury broadly resulted in microglial death at the center sites; the absence of cells at the center sites allows for microglial proliferation for repopulation, whereas we found that microglial death or apoptosis did not appear during the hyperacute and acute phases of HIE. The specific mechanisms behind microglial proliferation in HIE require further investigation. Recent studies have shown that HDAC3 mediates proinflammatory microglial proliferation without affecting anti‐inflammatory microglia, suggesting that further exploration of HDAC3 and other factors in HIE is warranted [46].

We identified microglial subtypes (MG7, MG8, MG9, and MG11) expressing markers associated with astrocytes, neuronal, RBC, and OPC. Intriguingly, other studies have similarly observed microglial subsets with neuronal mRNA [47, 48]. It remains unclear whether these neuronal gene‐enriched microglia endogenously express neuronal genes or phagocytose neurons containing such mRNA. The neuronal gene‐enriched microglia increased after whisker deprivation [48]. We found that the increase of neuronal gene‐enriched microglia did not occur with the increase of phagocytic microglia, suggesting that neuronal gene‐enriched microglia may result from phagocytosis. However, the straight evidence needs to be further obtained. Notably, MHCII^high^ inflammatory microglia, related to the IFN I type signaling pathway, peak in the subacute phase of HIE. IFN‐I‐responsive microglia have been recognized recently, which engulf whole neurons and maintain excitatory/inhibitory balance during cortical development [48]. Our scRNA‐seq data also revealed that MHCII^high^ inflammatory microglia appear during brain development, which are largely boosted during the subacute phase of HIE. These microglia may be derived from phagocytic microglia, which is supported by the expression of the phagocytosis‐related gene *Cd68*. The findings that post‐phagocytosis macrophages turn to the inflammatory phenotype also support our opinion [49]. To further verify this evolution, microglia in culture expressed high levels of the MHC class II molecule and pro‐inflammatory factors such as IL‐1β and IL‐6.

A previous study has identified two neuroblast populations in the developing mouse brain, one expressing *Eomes*, *Tac2*, and *Calb2*, and the other expressing *Gal* [50]. Our study expands this to eleven neuroblast populations, including a distinct subset associated with HIE, characterized by metal ion‐related neurodegeneration and the expression of *Nrgn*, *Clu*, and *Apoe* [51]. Metal ions such as Fe, Cu, and Zn loading in the neuroblasts following HIE induced the generation of this population, which exhibited age‐inappropriate neurodegeneration. However, the detailed mechanism needs to be further explored. OPCs were yet affected at the hyperacute stage of HIE, which were defined as ischemia‐responsible OPCs upregulating *Vgf*. The gene *Vgf* is NGF‐and BDNF‐inducible, encoding a 617‐amino‐acid precursor polypeptide. This precursor polypeptide is processed into some bioactive peptides that regulate neuronal survival and microglial phagocytose [52, 53]. However, the function of *Vgf* in OPCs following HIE deserves exploring. OCs exhibit a delayed response post‐HIE, with antigen presentation‐related OCs potentially inducing adaptive immunity and ER stress‐related OCs leading to apoptosis [54].

Progressive acquired hydrocephalus of unknown origin was observed in HIE infants [55, 56]. Our data recognized a novel LEC associated with senescence following HIE, which may impair cerebrospinal fluid lymphatic drainage and contribute to hydrocephalus. Investigating anti‐aging agents and the roles and mechanisms of these LECs could be beneficial. Fibroblast growth factors (FGFs), such as FGF‐10, FGF‐2, and FGF‐21, are known to promote neurological recovery following HIE, but the role of fibroblasts in HIE remains unclear [57, 58, 59]. Our findings indicate that fibroblasts are primarily altered during the subacute phase of HIE, potentially involved in brain remodeling.

Osteopontin (OPN), encoded by *Spp1*, is a cytokine‐like glycoprotein that binds to integrins and CD44 and is upregulated in stroke, CNS injury, and neurodegeneration. It promotes inflammation and wound healing [60, 61]. During development, embryonic microglia participate in axon tract and limit the size of the microcavities depending on the expression of OPN [62]. In addition, some studies also defined *Spp1* ^+^ macrophages as profibrotic macrophages that activate myofibroblasts [63]. Our data reveal an expansion of *Spp1* ^+^ macrophages and fibroblasts following HIE, highlighting the need for further understanding of their roles and mechanisms.

## Author Contributions

J.W. designed this study. X.C. conducted sample collection and scRNA‐seq analysis. J.W. established HIE models. X.C. and X.T. helped with the scRNA‐seq analysis. X.C. wrote the manuscript. J.W. modified the manuscript.

## Conflicts of Interest

The authors declare no conflicts of interest.

## Supporting information


**Figure S1:** UMAP plots showing brain major cell types from each group.

**Figure S2:** Enrichment pathway analysis of astrocyte DEGs at 3 h (A), 2d (B), and 7d (C) after HIE.

**Figure S3:** Enrichment pathway analysis of DEGs of A5 (A) and A7 (B).

**Figure S4:** Identification of astrocyte subpopulations after HIE. (A and B) Enrichment pathway analysis of sig. DEGs of A6 (A) and A7 (B). (C) UMAP plots showing astrocyte subtypes from each group.

**Figure S5:** A heatmap plot showing the top 10 DEGs expressed in each microglia subpopulation.

**Figure S6:** Identification of microglial subpopulations after HIE. (A) UMAP plots showing microglial subtypes from each group. (B) Pathway enrichment analysis of DEGs of MG6. (C) CytoTRACE analysis showing maturity of each microglial subtype.

**Figure S7:** Identification of neuroblast and oligodendrocyte lineage subpopulations after HIE. (A) A dot plot showing the top 5 DEGs expressed in each neuroblast subpopulation. (B) UMAP plots showing neuroblast subtypes from each group. (C) Enrichment pathway analysis of NB3 significant DEGs. (D) Enrichment pathway analysis of NB5 and NB11.

**Figure S8:** Identification of neuroblast and oligodendrocyte lineage subpopulations after HIE. (A) Enrichment pathway analysis of OPC1, 2, 5, and 6 significant DEGs, respectively. (B) UMAP plots showing oligodendrocyte lineage subtypes from each group.

**Figure S9:** STRING analysis of gene–gene interaction network of OPC5 DEGs.

**Figure S10:** Identification of endotheliocyte and fibroblast lineage subpopulations after HIE. (A) A dot plot showing the top 5 DEGs expressed in each endotheliocyte subpopulation. (B) UMAP plots showing endotheliocyte subtypes from each group. (C) Enrichment pathway analysis of LEC‐valve3 significant DEGs. (D) UMAP plots showing fibroblast subtypes from each group.

**Figure S11:** Identification of infiltrated immune cell subpopulations after HIE. (A) UMAP plots showing infiltrated immune cell subtypes from each group. (B) A Venn diagram showing the overlap genes between B cell‐A type and B type. (C and D) Enrichment pathway analysis of B cell‐A type (C) and B type (D) significant DEGs, respectively.

**Table S1:** fsb270929‐sup‐0012‐TableS1.xlsx.

**Table S2:** fsb270929‐sup‐0013‐TableS2.xlsx.

**Table S3:** fsb270929‐sup‐0014‐TableS3.xlsx.

**Table S4:** fsb270929‐sup‐0015‐TableS4.xlsx.

**Table S5:** fsb270929‐sup‐0016‐TableS5.xlsx.

**Table S6:** fsb270929‐sup‐0017‐TableS6.xlsx.

**Table S7:** fsb270929‐sup‐0018‐TableS7.xlsx.

**Table S8:** fsb270929‐sup‐0019‐TableS8.xlsx.

**Table S9:** fsb270929‐sup‐0020‐TableS9.xlsx.

**Table S10:** fsb270929‐sup‐0021‐TableS10.xlsx.

**Table S11:** fsb270929‐sup‐0022‐TableS11.xlsx.

## Data Availability

The raw data files of scRNA‐seq associated with this study have been deposited in the Gene Expression Omnibus under the number GSE273044. Additional data can be requested from the corresponding author.
