# Supplementary figures and images for: Single‐Cell Transcriptomic Profiling of Brain Cells in Newborn Rats Following Hypoxic Ischemic Encephalopathy
Source: FASEB J. 2025 Aug 13;39(16):e70929. doi: 10.1096/fj.202402891RR (PMC12344624; doi:10.1096/fj.202402891RR)

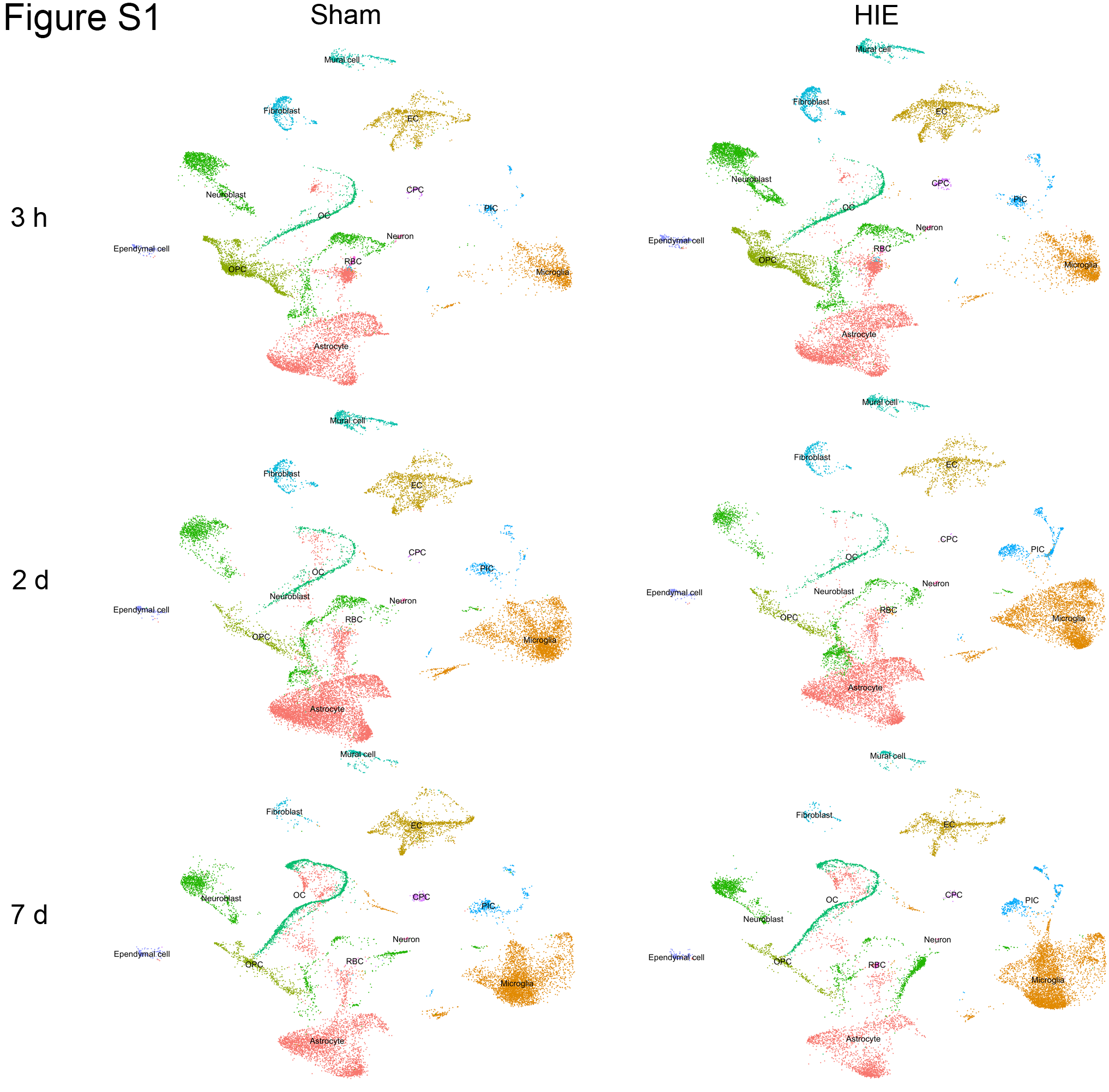

Supplement: Supplementary file 1 — Figure S1: UMAP plots showing brain major cell types from each group. [file FSB2-39-e70929-s022.tif]

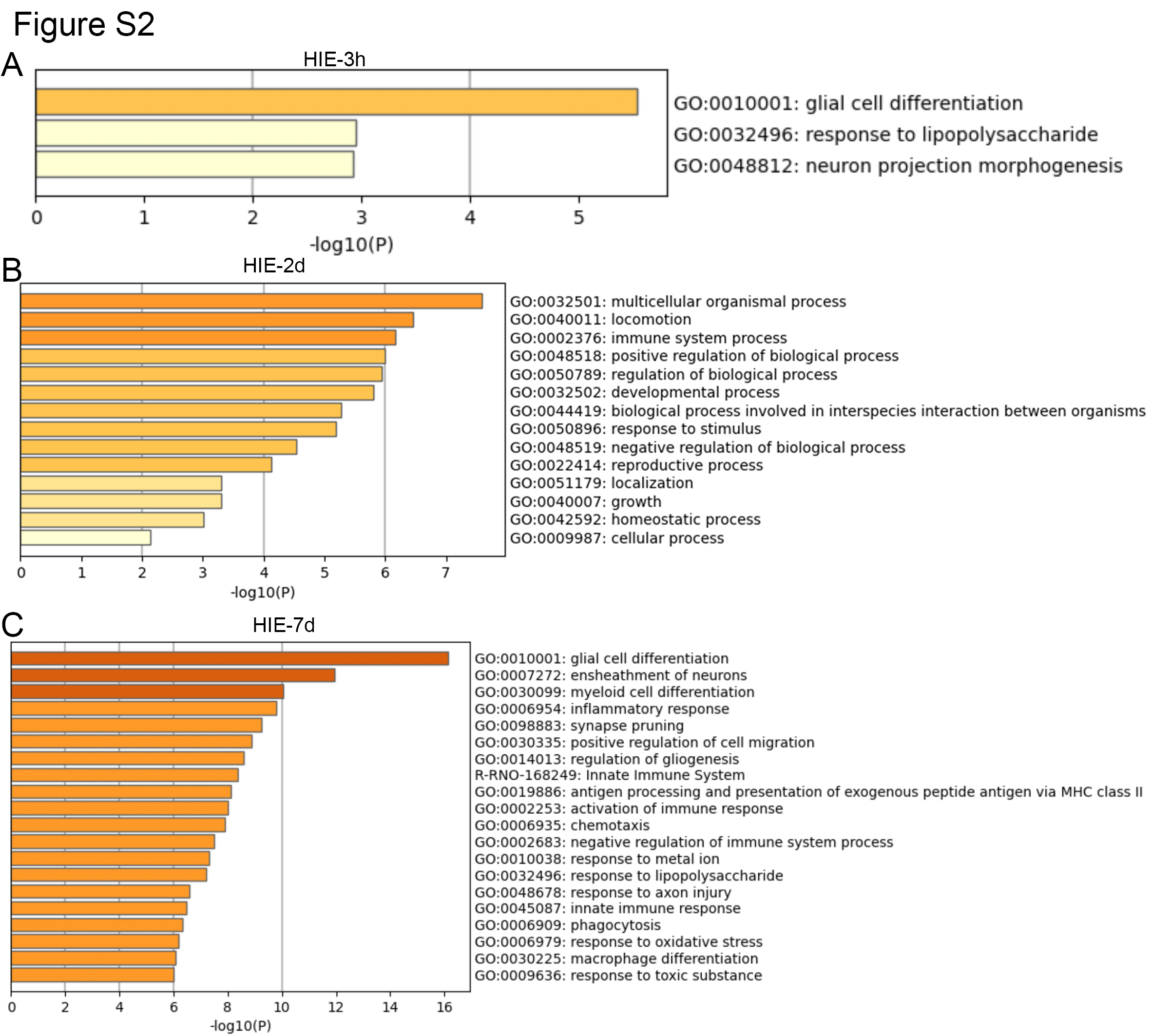

Supplement: Supplementary file 2 — Figure S2: Enrichment pathway analysis of astrocyte DEGs at 3 h (A), 2d (B), and 7d (C) after HIE. [file FSB2-39-e70929-s002.tif]

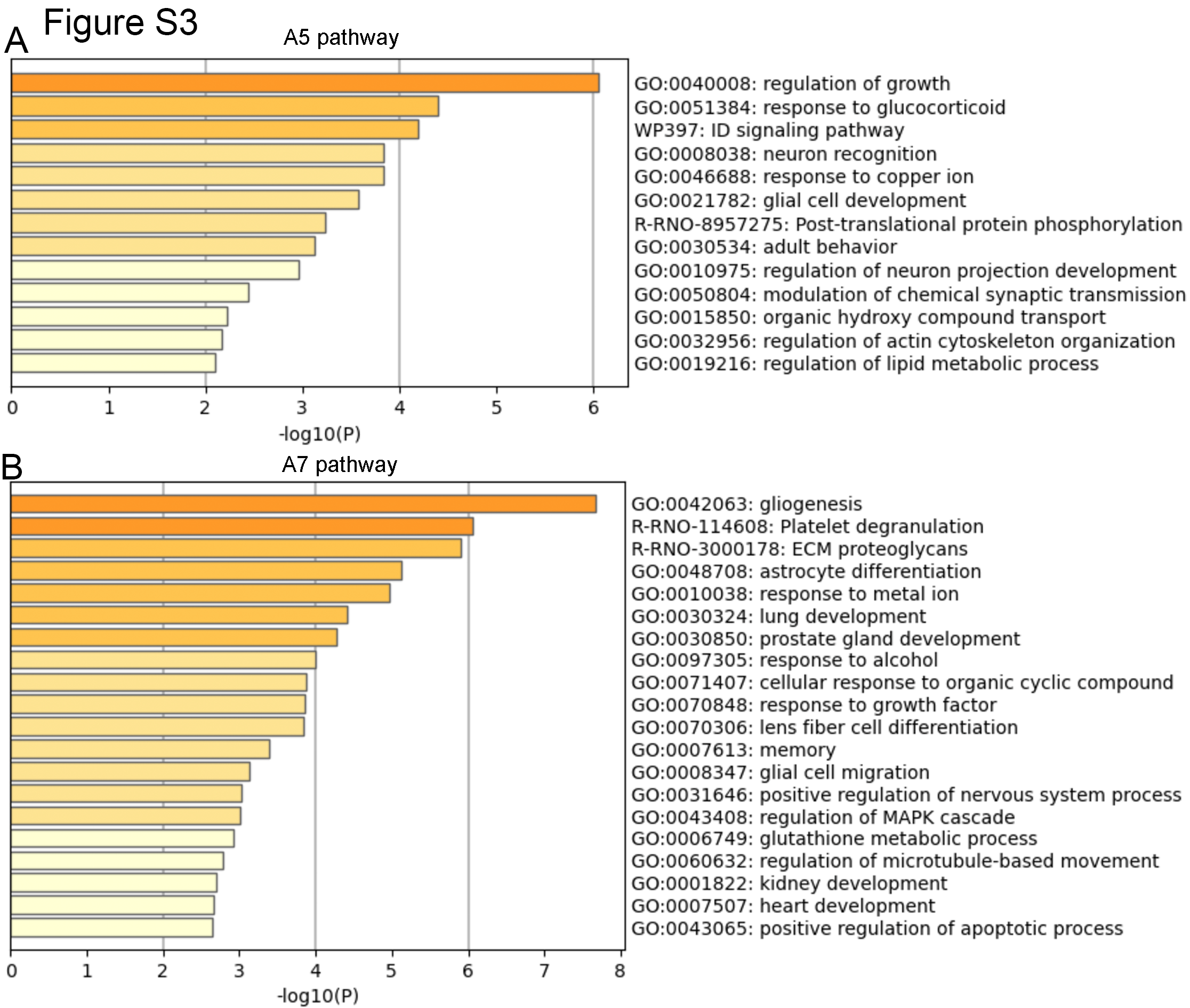

Supplement: Supplementary file 3 — Figure S3: Enrichment pathway analysis of DEGs of A5 (A) and A7 (B). [file FSB2-39-e70929-s009.tif]

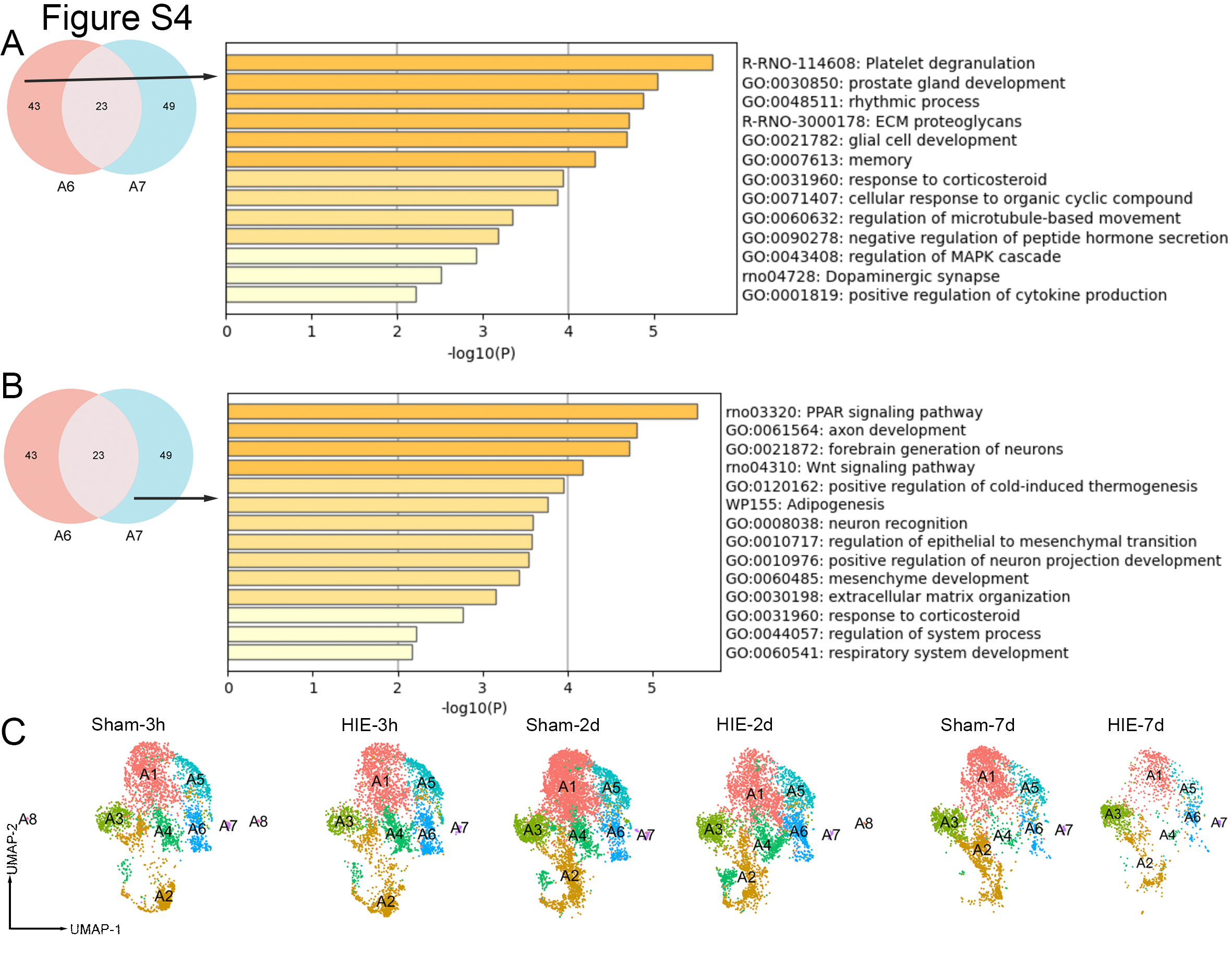

Supplement: Supplementary file 4 — Figure S4: Identification of astrocyte subpopulations after HIE. (A and B) Enrichment pathway analysis of sig. DEGs of A6 (A) and A7 (B). (C) UMAP plots showing astrocyte subtypes from each group. [file FSB2-39-e70929-s005.tif]

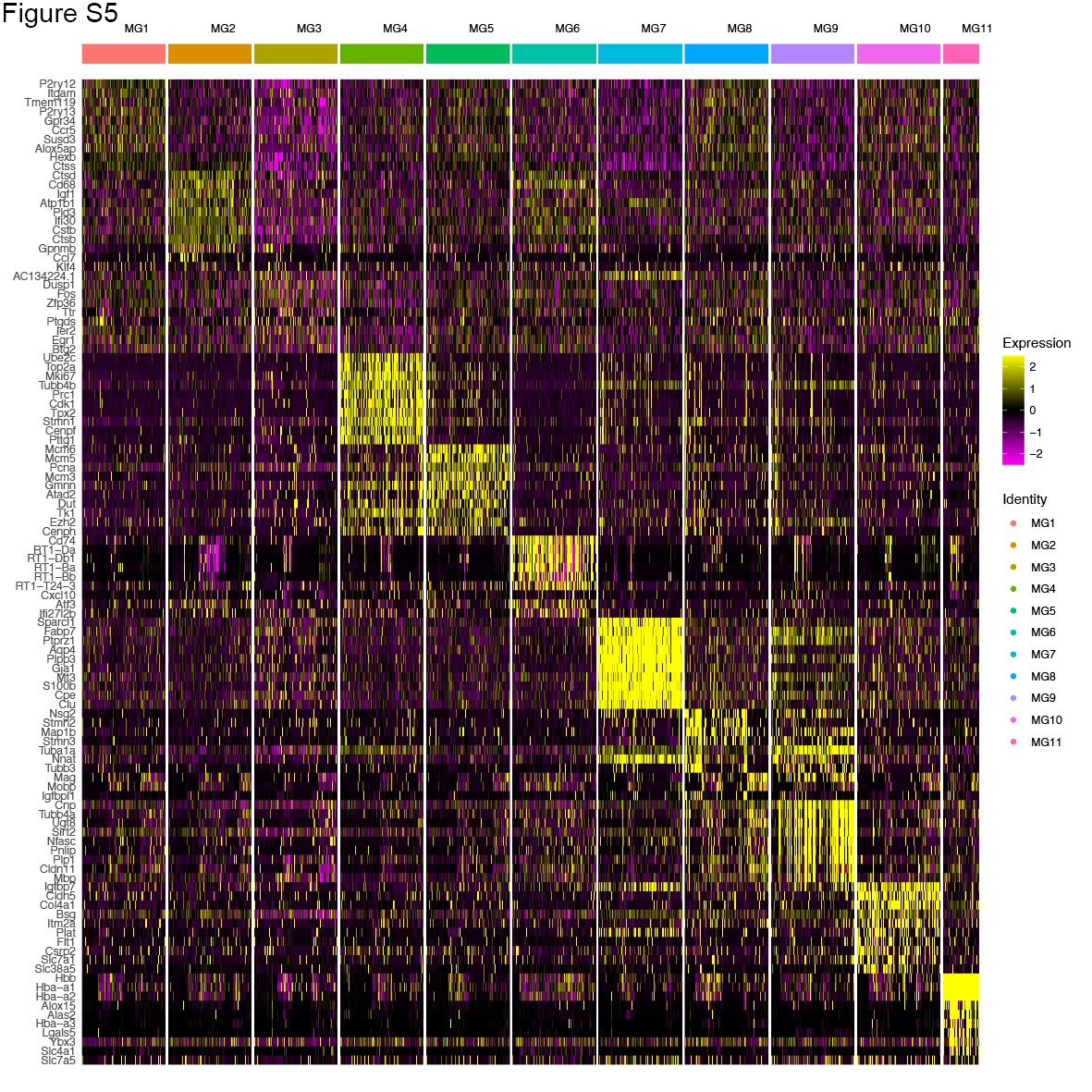

Supplement: Supplementary file 5 — Figure S5: A heatmap plot showing the top 10 DEGs expressed in each microglia subpopulation. [file FSB2-39-e70929-s019.tif]

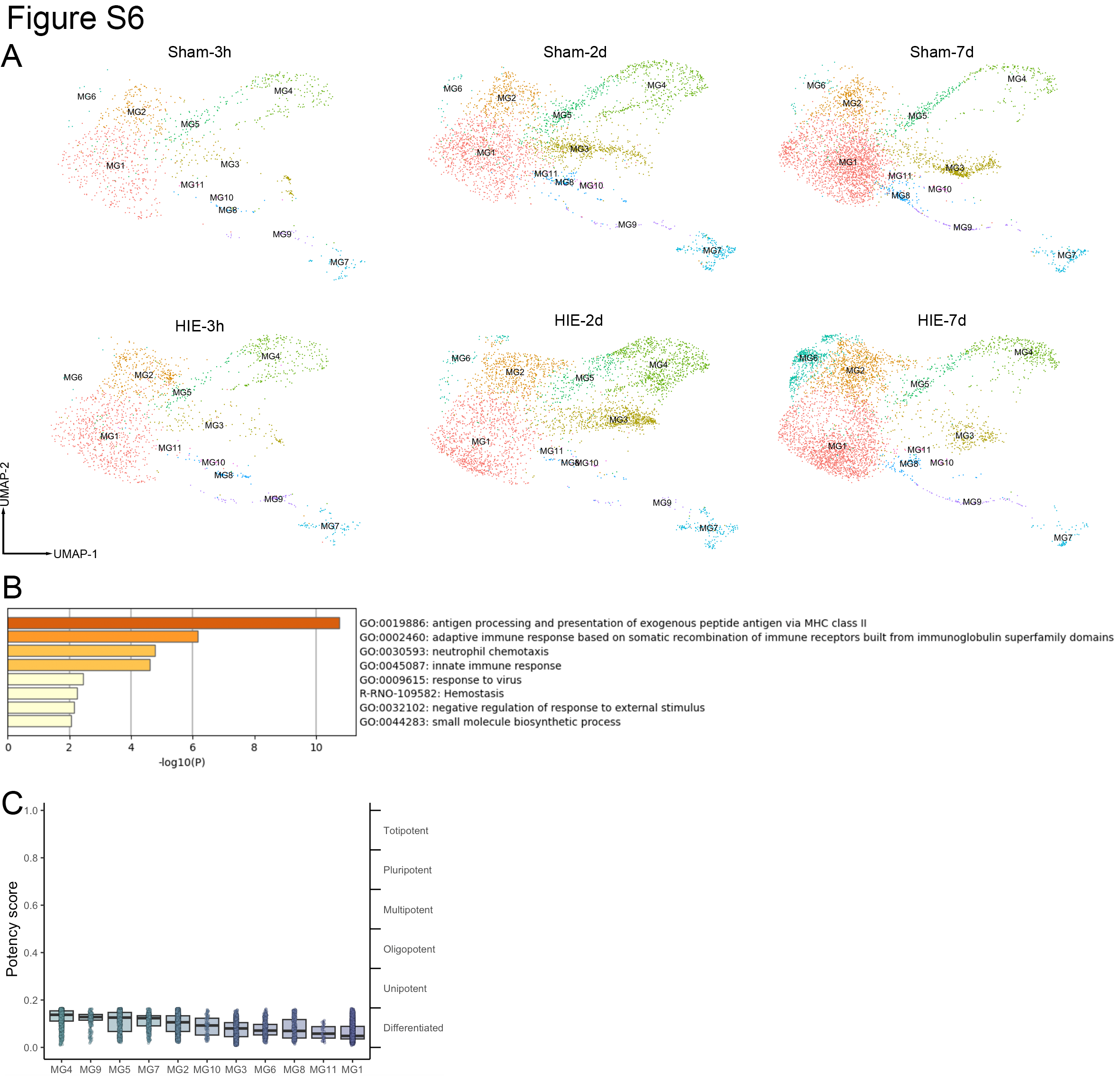

Supplement: Supplementary file 6 — Figure S6: Identification of microglial subpopulations after HIE. (A) UMAP plots showing microglial subtypes from each group. (B) Pathway enrichment analysis of DEGs of MG6. (C) CytoTRACE analysis showing maturity of each microglial subtype. [file FSB2-39-e70929-s017.tif]

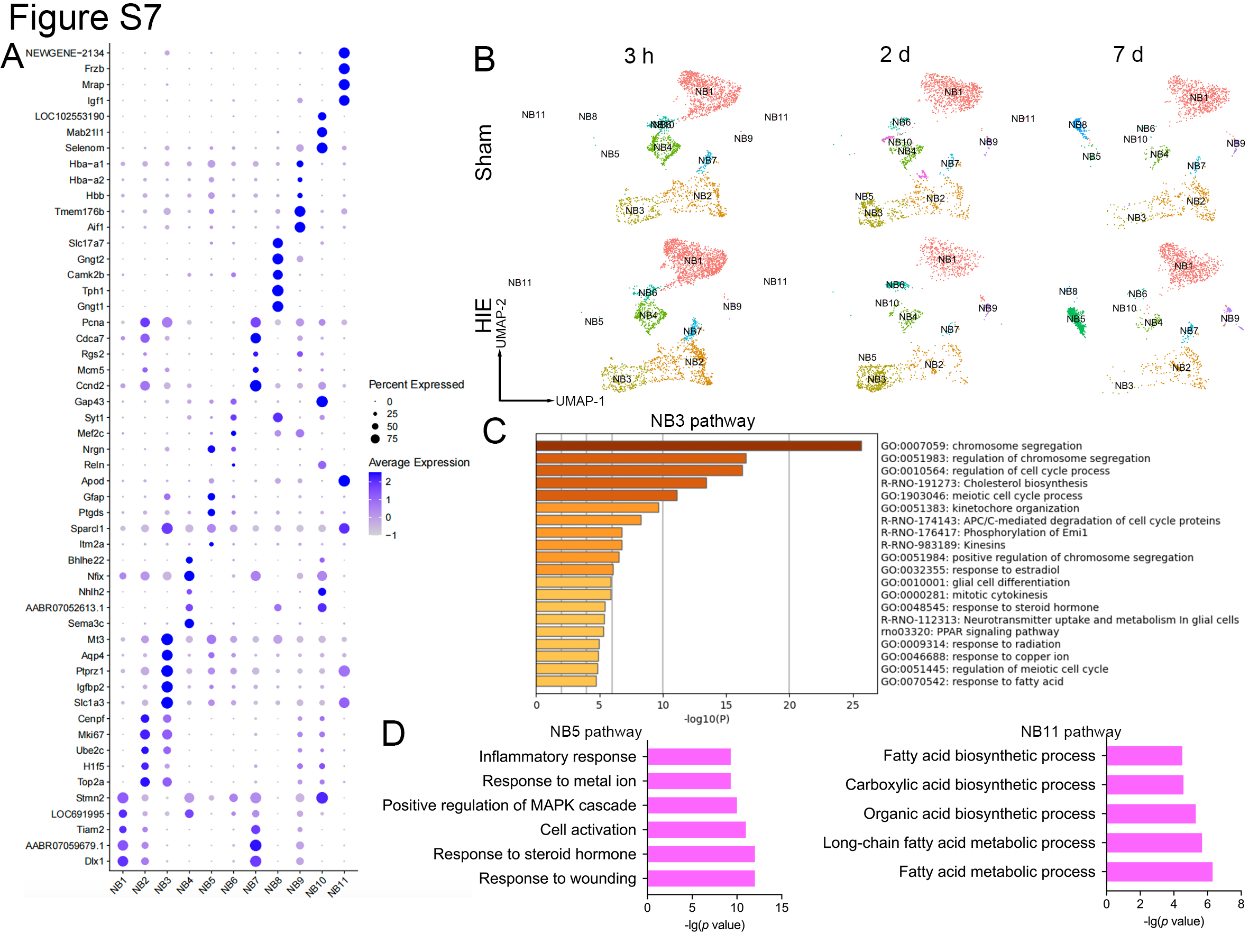

Supplement: Supplementary file 7 — Figure S7: Identification of neuroblast and oligodendrocyte lineage subpopulations after HIE. (A) A dot plot showing the top 5 DEGs expressed in each neuroblast subpopulation. (B) UMAP plots showing neuroblast subtypes from each group. (C) Enrichment pathway analysis of NB3 significant DEGs. (D) Enrichment pathway analysis of NB5 and NB11. [file FSB2-39-e70929-s008.tif]

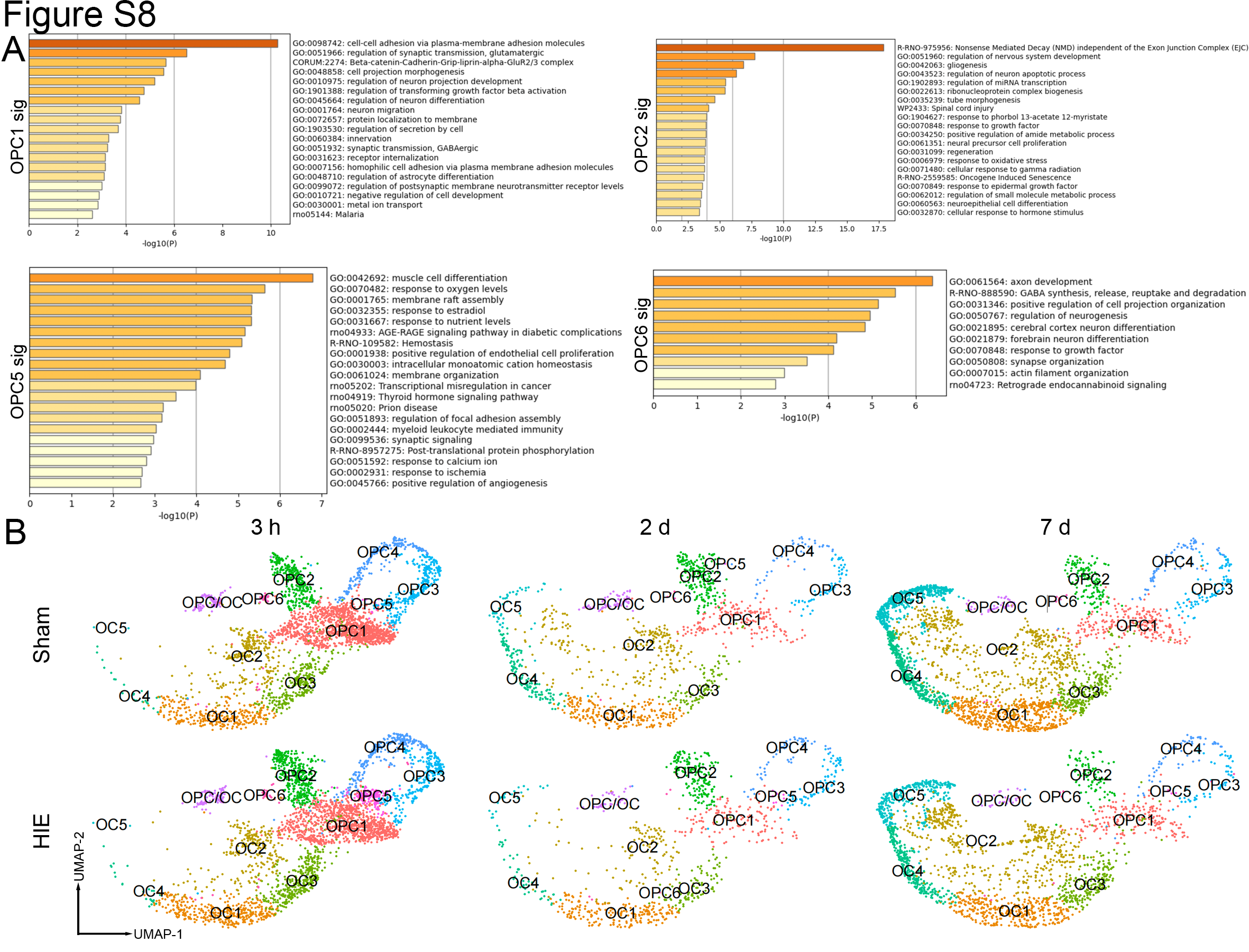

Supplement: Supplementary file 8 — Figure S8: Identification of neuroblast and oligodendrocyte lineage subpopulations after HIE. (A) Enrichment pathway analysis of OPC1, 2, 5, and 6 significant DEGs, respectively. (B) UMAP plots showing oligodendrocyte lineage subtypes from each group. [file FSB2-39-e70929-s012.tif]

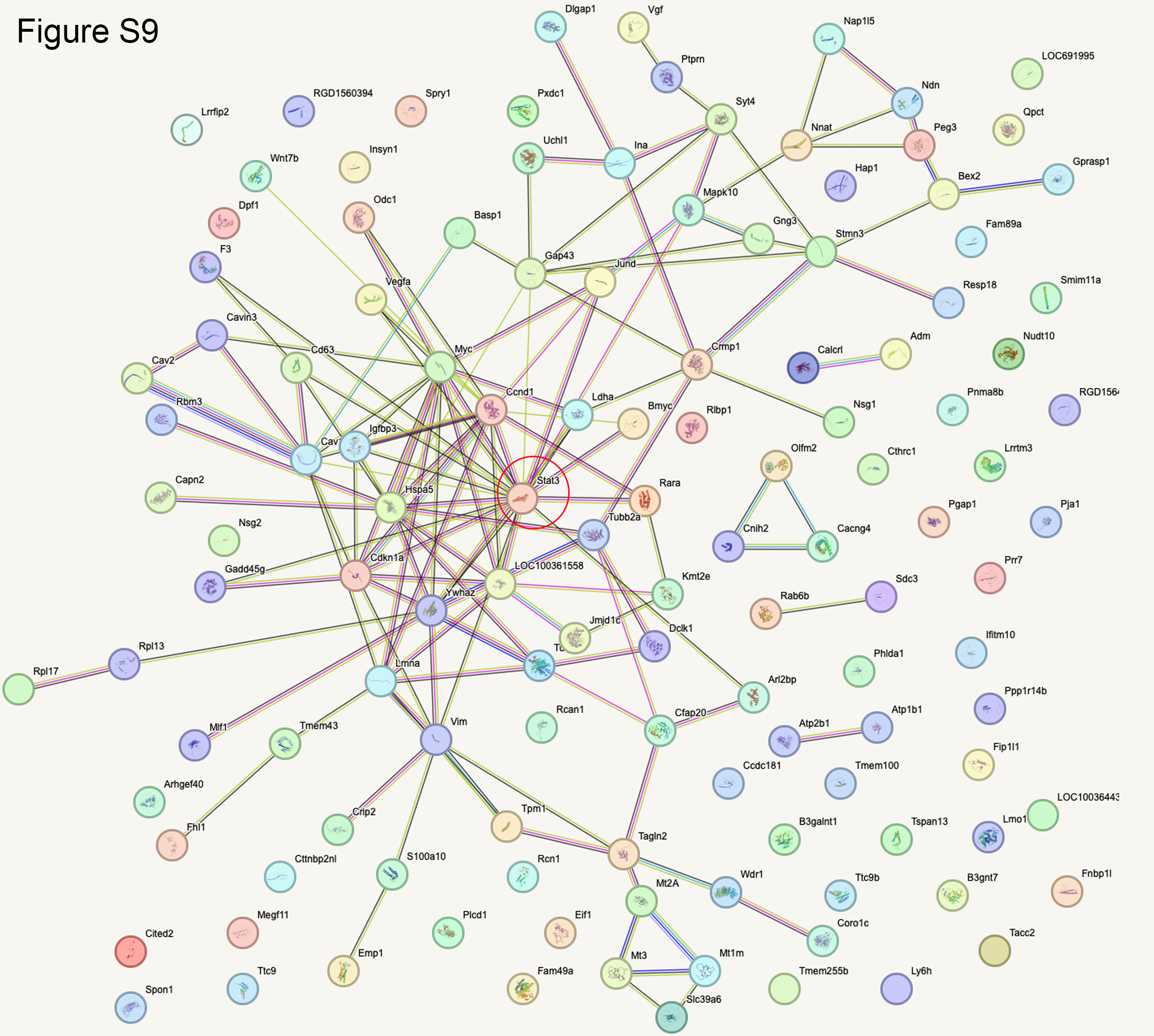

Supplement: Supplementary file 9 — Figure S9: STRING analysis of gene–gene interaction network of OPC5 DEGs. [file FSB2-39-e70929-s021.tif]

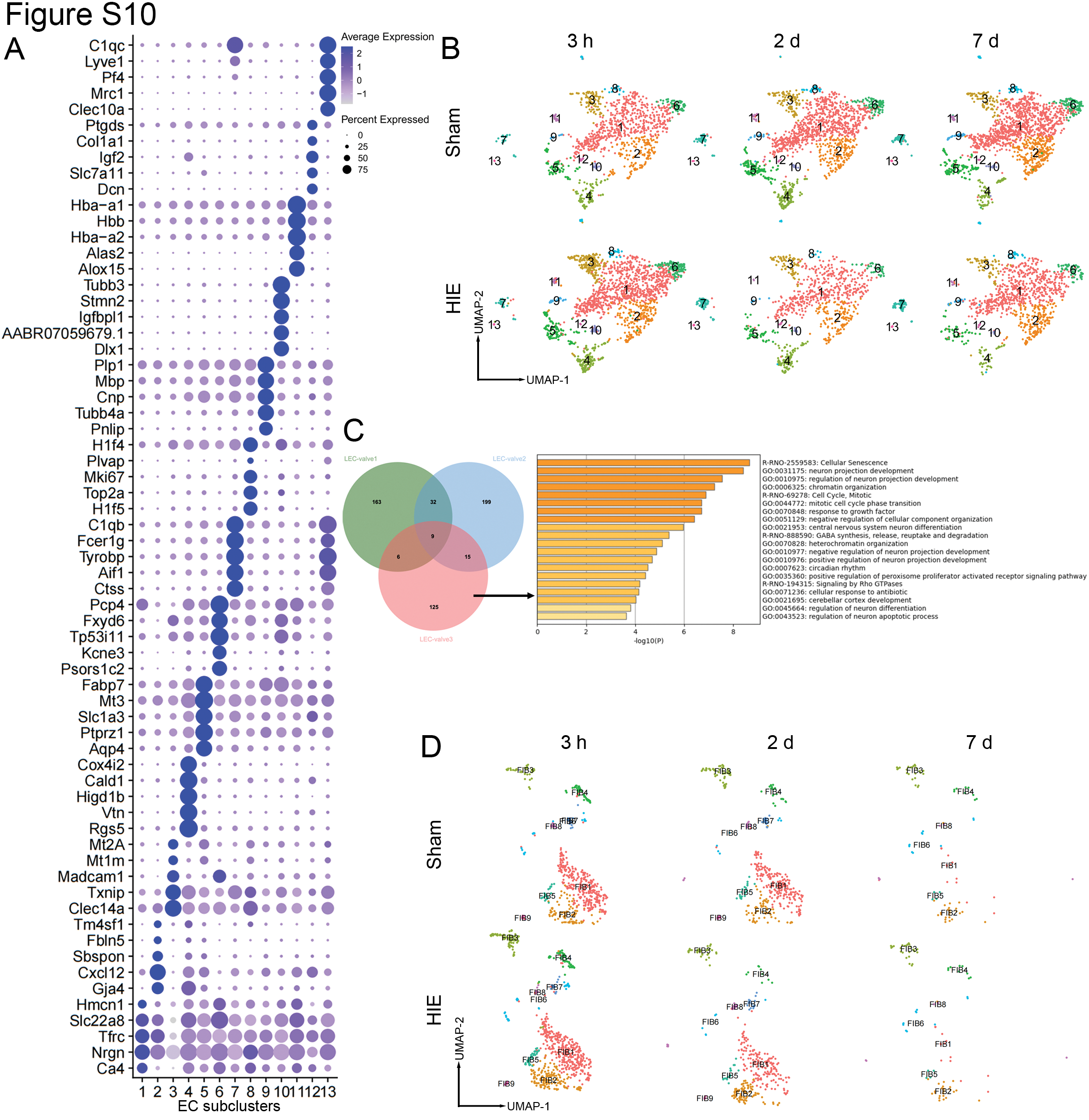

Supplement: Supplementary file 10 — Figure S10: Identification of endotheliocyte and fibroblast lineage subpopulations after HIE. (A) A dot plot showing the top 5 DEGs expressed in each endotheliocyte subpopulation. (B) UMAP plots showing endotheliocyte subtypes from each group. (C) Enrichment pathway analysis of LEC‐valve3 significant DEGs. (D) UMAP plots showing fibroblast subtypes from each group. [file FSB2-39-e70929-s011.tif]

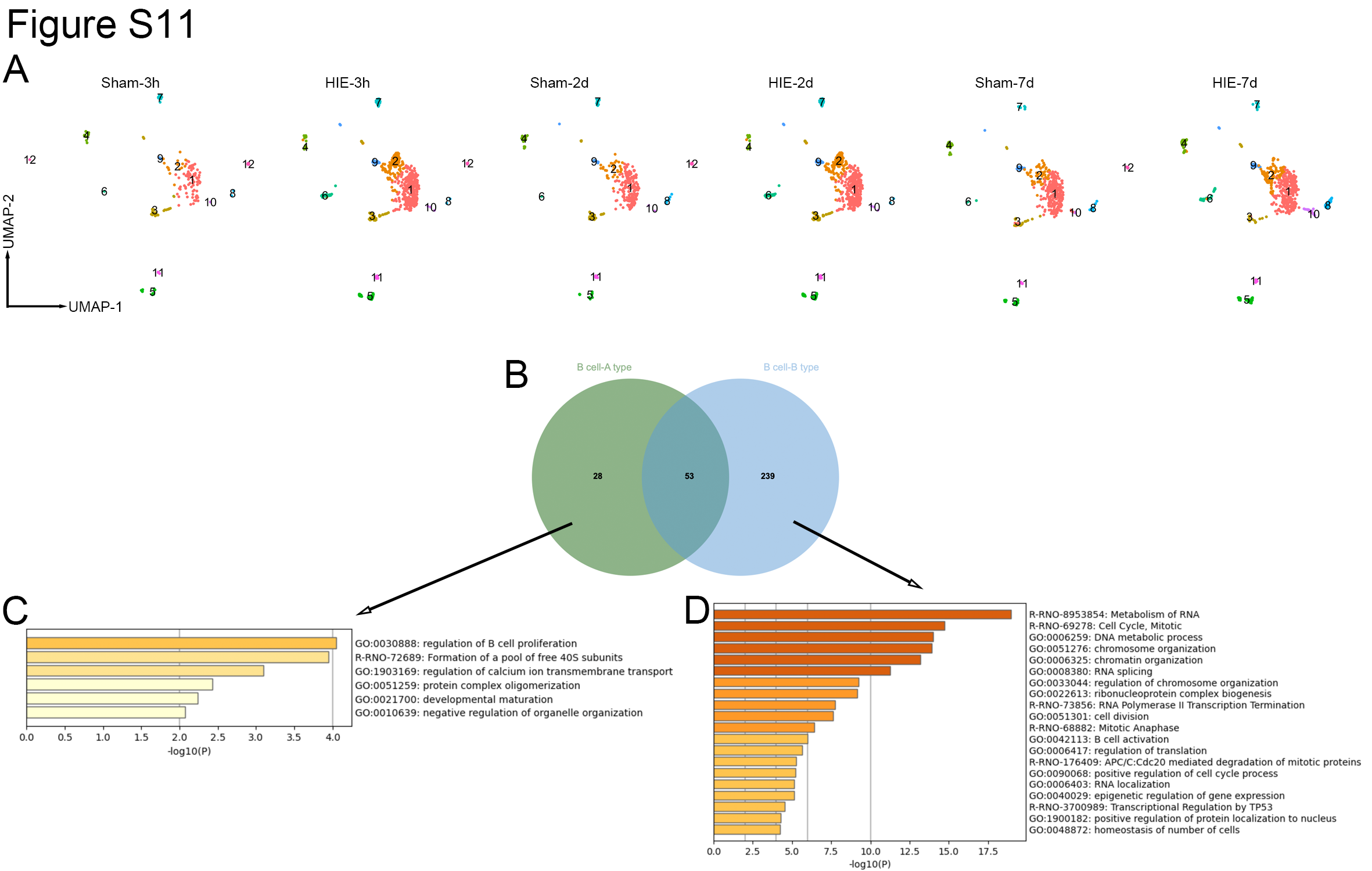

Supplement: Supplementary file 11 — Figure S11: Identification of infiltrated immune cell subpopulations after HIE. (A) UMAP plots showing infiltrated immune cell subtypes from each group. (B) A Venn diagram showing the overlap genes between B cell‐A type and B type. (C and D) Enrichment pathway analysis of B cell‐A type (C) and B type (D) significant DEGs, respectively. [file FSB2-39-e70929-s001.tif]
